# NGS in Hereditary Ataxia: When Rare Becomes Frequent

**DOI:** 10.3390/ijms22168490

**Published:** 2021-08-06

**Authors:** Daniele Galatolo, Giovanna De Michele, Gabriella Silvestri, Vincenzo Leuzzi, Carlo Casali, Olimpia Musumeci, Antonella Antenora, Guja Astrea, Melissa Barghigiani, Roberta Battini, Carla Battisti, Caterina Caputi, Ettore Cioffi, Giuseppe De Michele, Maria Teresa Dotti, Tommasina Fico, Chiara Fiorillo, Serena Galosi, Maria Lieto, Alessandro Malandrini, Marina A. B. Melone, Andrea Mignarri, Gemma Natale, Elena Pegoraro, Antonio Petrucci, Ivana Ricca, Vittorio Riso, Salvatore Rossi, Anna Rubegni, Arianna Scarlatti, Francesca Tinelli, Rosanna Trovato, Gioacchino Tedeschi, Alessandra Tessa, Alessandro Filla, Filippo Maria Santorelli

**Affiliations:** 1 Molecular Medicine for Neurodegenerative and Neuromuscular Diseases Unit, IRCCS Stella Maris Foundation, 56128 Pisa, Italy; daniele.galatolo1408@gmail.com (D.G.); gastrea@fsm.unipi.it (G.A.); mely.b91@hotmail.com (M.B.); roberta.battini@fsm.unipi.it (R.B.); Gemmanatale@yahoo.it (G.N.); ricca.ivana@gmail.com (I.R.); anna.rubegni@gmail.com (A.R.); ariannascarlatti@outlook.it (A.S.); f.tinelli@fsm.unipi.it (F.T.); rosy.tr@hotmail.it (R.T.); 2 Department of Neurosciences, Reproductive and Odontostomatological Sciences, Federico II University, 80131 Naples, Italy; giodemic@gmail.com (G.D.M.); antonella.antenora@virgilio.it (A.A.); giuseppe.demichele@unina.it (G.D.M.); fico.tina@gmail.com (T.F.); lietomaria@gmail.com (M.L.); 3 UOC Neurologia, Fondazione Policlinico Universitario ‘A. Gemelli’ IRCCS, 00168 Rome, Italy; gabriella.silvestri@unicatt.it (G.S.); vriso90@gmail.com (V.R.); salvatorerossi309@gmail.com (S.R.); 4 Department of Neurosciences, Università Cattolica del Sacro Cuore, 00168 Rome, Italy; 5 Department of Human Neuroscience, Sapienza University of Rome, 00185 Rome, Italy; vincenzo.leuzzi@uniroma1.it (V.L.); caterina.caputi@uniroma1.it (C.C.); serena.galosi@uniroma1.it (S.G.); 6 Department of Medical and Surgical Sciences and Biotechnologies, Sapienza University of Rome, 40100 Latina, Italy; Carlo.Casali@uniroma1.it (C.C.); ettore.snake@gmail.com (E.C.); 7 Unit of Neurology and Neuromuscular Disorders, Department of Clinical and Experimental Medicine, University of Messina, 98122 Messina, Italy; olimpia.musumeci@unime.it; 8 Department of Medicine, Surgery and Neurosciences, University of Siena, 53100 Siena, Italy; carla.battisti@unisi.it (C.B.); maria.dotti@unisi.it (M.T.D.); alessandro.malandrini@unisi.it (A.M.); andrea.mignarri@teletu.it (A.M.); 9 Paediatric Neurology and Muscular Diseases Unit, University of Genoa and ‘G. Gaslini’ Institute, 16147 Genoa, Italy; chi.fiorillo@gmail.com; 10 Center for Rare Diseases and Interuniversity Center for Research in Neurosciences, Department of Advanced Medical and Surgical Sciences, 2nd Division of Neurology, University of Campania “Luigi Vanvitelli”, 80131 Naples, Italy; marina.melone@unicampania.it (M.A.B.M.); gioacchino.tedeschi@unicampania.it (G.T.); 11 Department of Neurosciences, University of Padua, 35121 Padua, Italy; elena.pegoraro@unipd.it; 12 Azienda Ospedaliera San Camillo-Forlanini, 00152 Rome, Italy; anpetrucci@scamilloforlanini.rm.it

**Keywords:** HA, next-generation sequencing, cohort, targeted resequencing panel, TRP, exome sequencing, diagnostic yield, variant, mutation, Genesis

## Abstract

The term hereditary ataxia (HA) refers to a heterogeneous group of neurological disorders with multiple genetic etiologies and a wide spectrum of ataxia-dominated phenotypes. Massive gene analysis in next-generation sequencing has entered the HA scenario, broadening our genetic and clinical knowledge of these conditions. In this study, we employed a targeted resequencing panel (TRP) in a large and highly heterogeneous cohort of 377 patients with a clinical diagnosis of HA, but no molecular diagnosis on routine genetic tests. We obtained a positive result (genetic diagnosis) in 33.2% of the patients, a rate significantly higher than those reported in similar studies employing TRP (average 19.4%), and in line with those performed using exome sequencing (ES, average 34.6%). Moreover, 15.6% of the patients had an uncertain molecular diagnosis. *STUB1*, *PRKCG*, and *SPG7* were the most common causative genes. A comparison with published literature data showed that our panel would have identified 97% of the positive cases reported in previous TRP-based studies and 92% of those diagnosed by ES. Proper use of multigene panels, when combined with detailed phenotypic data, seems to be even more efficient than ES in clinical practice.

## 1. Introduction

The term hereditary ataxia (HA) refers to a heterogeneous group of rare neurodegenerative disorders with a wide spectrum of ataxia-dominated phenotypes. Gait abnormalities, lack of coordination, dysarthria, and dysmetria are the most common clinical traits, associated with degeneration of Purkinje cells and/or spinocerebellar connections, often combined with atrophy of other regions of both the central and peripheral nervous systems [1].

Although these conditions have been formally classified on the basis of patterns of transmission and disease–gene relationships, different examples of commonalities with a range of clinical syndromes are now rapidly emerging. As a result, it is common to see HA overlapping with other neurological diseases (e.g., hereditary spastic paraplegia, epilepsy, and hypo- and hyperkinetic movement disorders) [2]. Autosomal dominant spinocerebellar ataxia (SCA) currently has an overall estimated prevalence of 1.5–4.0 × 10^−5^ [3] and includes more than 40 clinical conditions [4]. Most of these, caused by pathological trinucleotide repeat expansions in coding regions, are termed polyQ SCA. On the other hand, the forms collectively termed autosomal recessive spinocerebellar ataxia (SCAR, prevalence: 1.8–4.9 × 10^−5^ [3]) are caused by mutations in more than 100 genes. Changes in a similar number of genes are responsible for recessive forms in which ataxia is only part of the clinical picture.

Thanks to the advent and growth of next-generation sequencing (NGS), our knowledge of HA and its genetic pathogenesis has broadened over the past decade [5]. Since its first application in a small subset of patients [6], various—now common—clinical applications of the technique (i.e., based on targeted resequencing panels (TRPs) and exome sequencing (ES)) have been used in attempts to establish genetic diagnoses in several cohorts with undetermined ataxia. Overall, TRP and ES have been found to have an average diagnostic yield of 19.4% and 34.6%, respectively. However, because of differences in gene coverage, performance quality, data analysis, and global costs, together with the different needs of specific laboratories, it remains difficult to identify the most powerful technique in absolute terms.

Here, we report a large cross-sectional study of 377 highly heterogeneous patients with genetically uncharacterized HA. Using a TRP approach, we obtained a diagnostic rate of 33.2%., nearly twice that reported in other TRP-based studies and comparable to those employing ES. Overall, our data point to increasingly frequent mutation of genes until now considered only very rarely involved in HA and show that massive parallel sequencing is currently unveiling a large set of phenotypes associated with ataxia. They also indicate that TRPs are still suitable for the genetic screening of large cohorts of patients.

## 2. Results

**Figure 1:**
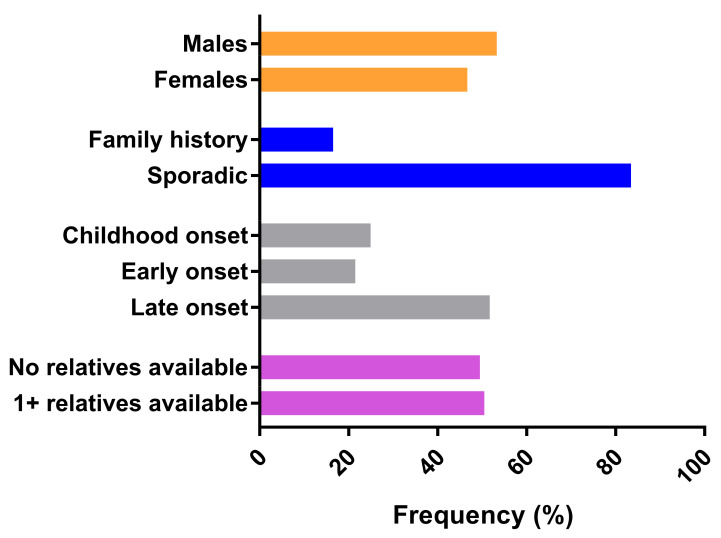
Features of the initial cohort, including the possibility of performing segregation studies (violet).

We analyzed a total of 377 index cases (201 males, 53.3%; 176 females, 46.7%) (Figure 1). Table 1 summarizes their genetic results. Sequencing coverage was significatively high, indicating extremely high reliability of our results (read depths, i.e., minimum number of reads/% of analyzable target regions covered, were as follows: 1/99.95%, 10/99.80%, 20/99.62%, 50/99.05%, 100/97.23%, 200/88.58%, 500/81.65%, 1000/52.11%).

A family history was found in 62 (16.5%) of our patients (27 autosomal dominant and 35 autosomal recessive), while the vast majority (315, 83.5%) were sporadic (Figure 1). At least one relative could be tested in 150 (39.8%) of the index cases. Overall, we tested 297 affected or unaffected relatives for segregation studies (Figure 1).

**Figure 2:**
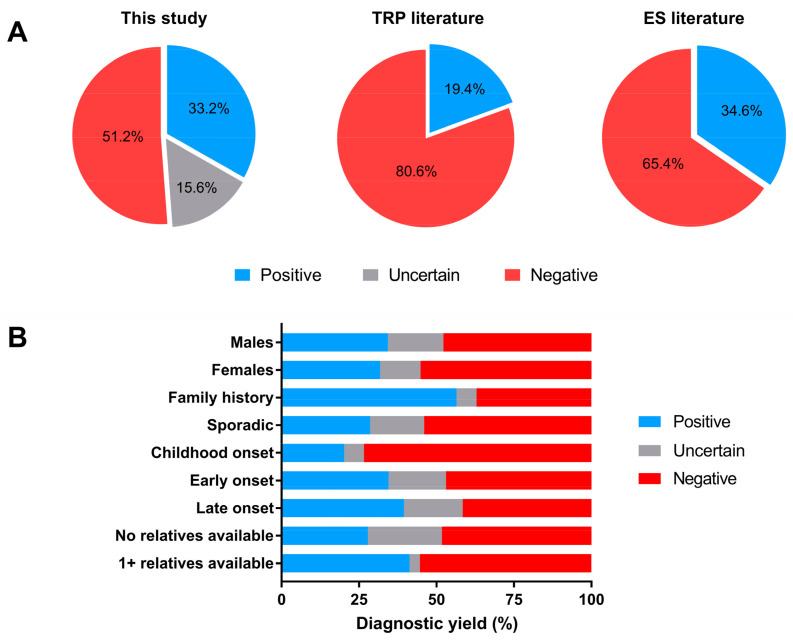
(**A**) Overall diagnostic yield achieved in this study compared with average results of TRP and ES approaches as described in the literature; (**B**) Diagnostic yield presented by specific features.

Phenotypically, our cohort was highly heterogeneous, including patients with pure cerebellar ataxia; spastic ataxia; congenital ataxia; sensory ataxia; and even ataxia with seizures, myoclonus, peripheral neuropathy, or combinations of these. This reflects the broad heterogeneity of HA seen in routine clinical practice in movement disorder centers and ataxia clinics. Their age at onset was also highly variable: we had childhood-/teenage-onset cases (<16 yrs; 94 index cases, 24.9%) as well as early- (<40 yrs; 81/377, 21.5%) and late-onset ones (≥40 yrs; 195/377, 51.7%); age at onset was not ascertained in seven patients (1.9%). The high level of clinical and genetic heterogeneity observed in this cohort is in line with routine referrals to neurogenetics laboratories in our country. Variants of pathogenic or putative pathogenic significance, defined according to the criteria of the American College of Medical Genetics and Genomics, were identified in 125 patients (33.2%) (Figure 2A): 69 males (55.2%) and 56 females (44.8%). Fifty-nine patients (15.6%) had an uncertain molecular diagnosis because of the presence of variants of unknown significance (VOUS). VOUS also included variants detected in patients with phenotypic data not detailed enough to help with variant prioritization and/or biallelic mutations that could not be phased due to lack of parental DNA (Figure 2A). Furthermore, patients assigned to the VOUS group also included those harboring variants of putative pathogenic significance in genes not clearly correlated with a specific phenotype, and those carrying single likely pathogenic variants in genes known to cause only recessive disorders [7]. One-hundred-and-ninety-three patients (51.2% of the whole set) were negative on NGS analysis (Figure 2A). It is tempting to advance various hypotheses in these cases: variants falling outside the coding exons of the genes included in our TRP strategy, variants in yet-to-be-discovered new genes or in genes not canonically associated with HA, or even the possibility of another condition mimicking HA.

In our study, the diagnostic yield in cases with a family history was twice that obtained in sporadic cases (Figure 2B). Moreover, the possibility of achieving a molecular diagnosis increased with increasing age at onset (Figure 2B). Unsurprisingly, the possibility to perform confirmatory genetic studies in other relatives also increased the diagnostic rate, whereas no significant differences were detected when considering the gender of the index cases (Figure 2B).

**Figure 3:**
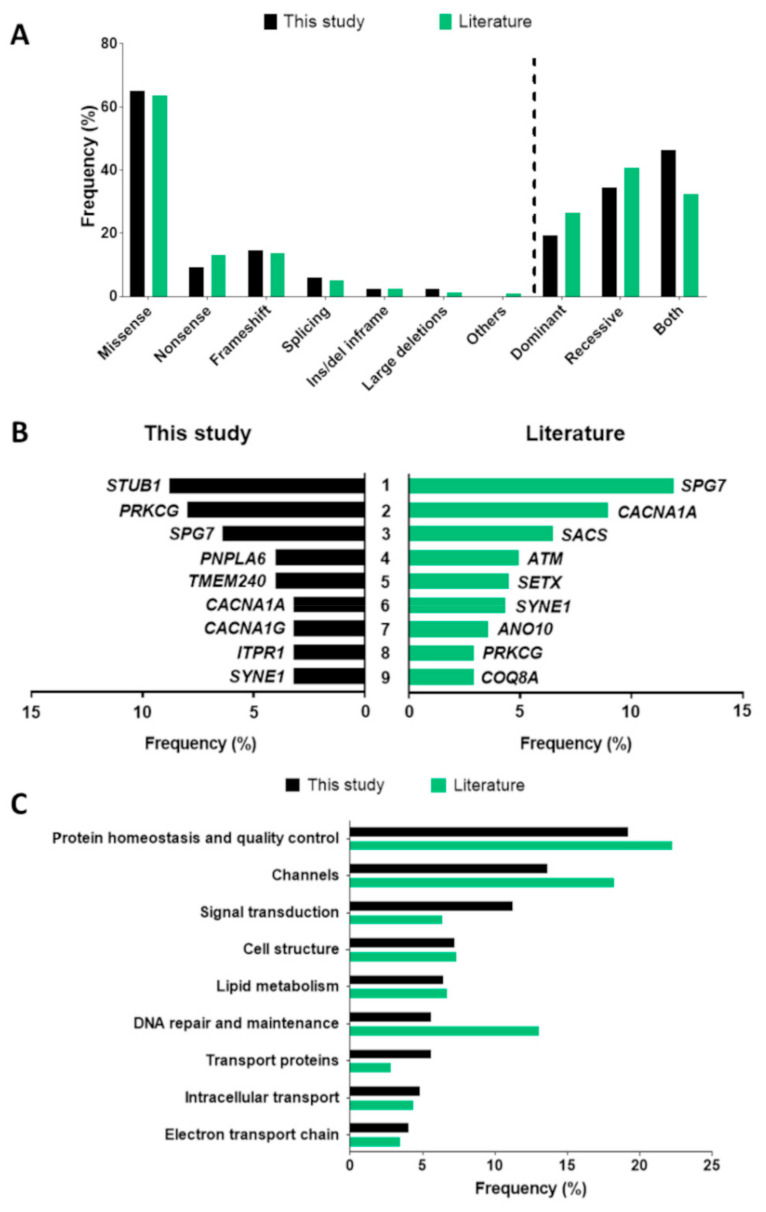
Comparison of molecular findings between this study (black) and literature (green) in terms of (**A**) mutation types and patterns of inheritance, (**B**) most common disease-causing genes, and (**C**) pathways involved.

Among the positive cases, our variant-filtering criteria identified 164 mutations as pathogenic or likely pathogenic (Table 1; *in silico* predictions, population frequencies, and ACMG classifications are listed in Table S1). Missense variants (107/164, 65.2%) were the most common, whereas frameshift, nonsense, splicing, large deletion, and in frame insertion or deletion mutations were less common, being found in 24 (14.6%), 15 (9.1%), 10 (6.1%), 4 (2.4%), and 4 (2.4%) patients, respectively (Figure 3A). Large deletions were identified on the basis of complete absence of coverage in the same *SPG7* exon in two cases, suggesting a possible homozygous single exon deletion subsequently confirmed by gene-specific multiplex ligation-dependent probe amplification (MLPA) testing.

To better define the pathogenic role of variants identified, we performed functional investigation *in silico* focusing on all novel missense variants of pathogenic significance (*n* = 36) detected. Phylogenetic examination showed that all variants are highly conserved through species (Figure S1), and protein domain localization analysis indicated that 28/36 (77.8%) mutations lay in regions supposed to have a critical role for protein function (Table S2). Moreover, to further confirm the robustness of our results, we took advantage of multiple computational methods to predict protein stability changes upon mutations in terms of changes in folding free energy (ΔΔG) between wild type and mutant structures. Among proteins whose 3D structures were freely available in online databases, we could computationally analyze the effect of seven variants of pathogenic significance. Together we analyzed four variants in *STUB1* found in this study and already known to be disease causing [61,63] used as positive controls. Our analyses converged to assess a destabilizing effect on protein stability in 4/7 cases (Table S3), providing changes in interatomic interactions (Figure S2).

Among the 125 cases with a positive diagnosis, we were able to study first-degree relatives in 62/125 (49.6%).

In 24/125 (19.2%) patients, SCA genes associated with non-polyQ forms were found to be causative, while SCAR genes were mutated in 40 (32.0%). Interestingly, nearly half of the positive index cases (58/125, 46.4%) harbored variants of pathogenic significance in genes not respecting zygosity rules and known to cause both SCA and SCAR (Figure 3A).

Variants in 56 different genes were considered causative in our group of 125 positive cases, indicating a very high level of genetic heterogeneity. Of note, the nine most common disease-causing genes (*STUB1*, *PRKCG*, *SPG7*, *CACNA1A*, *PNPLA6*, *SYNE1*, *TMEM240*, *CACNA1G*, and *ITPR1*, in that order of frequency) accounted for nearly half (55/125, 44.0%) of all our positive cases (Figure 3B). Thus, *STUB1* was the most common (11/125, 8.8%), followed by *PRKCG* (8.0%) and *SPG7* (6.4%) (Figure 3B). On sorting pathogenic variants by their associated GO terms and potential disease mechanisms, we observed that mutations in genes involved in protein homeostasis and quality control (found in 24/125 patients, 19.2%) were the most frequent in our cohort. However, genes coding for ion channels (17 index cases, 13.6%) or involved in signal transduction (14, 11.2%) were also frequent (Figure 3C). Furthermore, we noticed a significant frequency of variants in genes involved in cytoskeleton and cell ultrastructure functions (in nine index cases, 7.2%), lipid metabolism (eight, 6.4%), DNA repair and maintenance (seven cases, 5.6%), transport proteins (seven, 5.6%), intracellular transport (six, 4.8%), and electron respiratory chain/oxidative metabolism (five patients, 4.0%) (Figure 3C). Few patients had pathogenic variants in genes encoding tRNA synthetases, proteins involved in DNA replication and transcription, ciliary and mitochondrial biogenesis, and homeostasis; variants in genes linked to other molecular pathways were even rarer.

Among the 59 patients with an uncertain molecular diagnosis (Table S4), most had late-onset disease (>40 yrs; 37/59, 62.7%), almost all were sporadic cases (55/59, 93.2%), and in the majority it was not possible to study biological samples from relatives for segregation analyses (54, 91.5%). A positive family history and the possibility to perform segregation analysis can of course corroborate or exclude the pathogenic role of detected gene variants. Forty-seven of the 193 negative cases (24.3%) were initially considered as VOUS, but further assessments in family relatives helped to exclude the potential involvement of “candidate” variants.

We then compared our results with those published earlier by others. Our literature analysis (conducted using PubMed and Google Scholar; latest access 4 January 2021) identified 27 published studies that applied NGS methods in heterogeneous cohorts of genetically undiagnosed HA patients. TRP strategies were employed in nine (33%) studies and ES in 15 (56%), whereas three studies (11%) employed both methods (Table 2). Overall, the index cases involved in TRP and ES studies numbered 1262 and 1179, respectively.

In analyzing the literature data and calculating the weighted mean in each study in relation to its cohort size, we observed that the use of TRPs led to a mean diagnostic yield of 19.4% (range: 11–82%), whereas ES delivered a mean diagnostic rate of 34.6% (range: 20–57%) (Figure 2A). Table S5 lists the disease-causing genes in each study. We did not consider percentages of patients with VOUS, as this information is lacking in most studies. As in our study, the inclusion criteria used in previous reports analyzed following our literature search were broad, allowing the inclusion of pediatric and late-onset patients, familial or sporadic ones, and cases with different patterns of inheritance. In several studies (13/27, 48%), the inclusion criteria were even more relaxed, generating highly heterogenous cohorts. Interestingly, most studies suggest that in the presence of certain features, such as early onset, a positive family history, the availability of segregation studies, and consanguinity in the family, the likelihood of reaching a definitive genetic diagnosis is high.

With regard to the types of pathogenic variant reported in the literature (*n* = 847, Table S6), the overall data were similar to those of our study. Indeed, 63.7% of variants (538/847) were missense, 13.8% frameshift (117/847), 13.1% nonsense (111/847), 5% splicing (42/847), 2.5% ins/del inframe (21/847), and 1% large deletions (9/847), while polyQ expansions and mutations in mitochondrial DNA accounted for 0.8% (Figure 3A). When restricting the analysis to disease-causing genes found in positive cases (*n* = 649, Table S7), we observed relatively higher rates of SCAR (40.6%, 263) and non-polyQ SCA (26.5%, 172/649) than of forms showing both dominant and recessive patterns of inheritance (32.3%; 209/649). The rate of mtDNA mutations was much lower (0.6%, 5/649) (Figure 3A). *SPG7* was the most common disease-causing gene (accounting for 77 cases; 11.9%), followed by *CACNA1A* (58; 9%) and then *SACS* (42; 6.5%) (Figure 3B). Interestingly, the nine most frequent genes (*SPG7*, *CACNA1A*, *SACS*, *ATM*, *SETX*, *SYNE1*, *ANO10*, *PRKCG*, and *COQ8A*) were found to account for 50.5% (327/649) of all solved cases in the literature, a proportion close to the 43.5% found in our study (Figure 3B). Moreover, most (522/649, 80.4%) of the positive cases in literature were distributed among just 41 genes (likely the most common HA genes), with 100 rarer genes accounting for 122 cases (18.9%). Data suggest the existence of common, rare, and even ultra-rare HA genes. Apart from a few differences in terms of distribution, the most common biological pathways in our study reflected recent published data [94].

## 3. Discussion

The diagnostic rate (33.2%) obtained in our study was nearly twofold the average weighted value described in the TRP-based studies of HA (19.4%) reported in the literature, and comparable to the value reported in those using ES as a first-tier approach (34.6%). Considering the high coverage reached with multigene panels, and the easier and faster analysis of their results compared with the more commonly used ES method (where low, not always uniform coverage might result in gaps), it can be argued—as others have done [93]—that multigene panels are still worth using for quick screening of large cohorts. Our results indicate that the large multigene panel we designed would have intercepted 97.5% (235/241) of the diagnosed cases in published TRP series (excluding those with mtDNA mutations) and most of those solved by ES (92.2%; 376/408) (Table S8). The TRPs used in the literature included an average of 73 genes, and detected mutations in 45, whereas ES-based studies detected variants in 127 genes; these data further corroborate the high diagnostic power of our panel (>200 genes analyzable).

Nonetheless, with the costs of both techniques (ES in particular) now rapidly declining, we suggest that the most correct approach for the coming years—at least until we have cost-effective whole-genome sequencing (WGS) strategies suitable for routine clinical use—might be to combine ES (ensuring minimum average coverage of 100X) with an *in silico* panel (including the genes in our TRP) for gene prioritization.

It is likely to assume that the significantly higher diagnostic yield achieved in our study, compared to those described in other TRP-based approaches, mainly depends on the number of genes analyzed (285), that is four time higher than average value of panels in literature (73) and with the number of HA-related genes being increased over time. Indeed, despite only a dozen genes being responsible for the disease of half of our cohort, the genetic etiology of the half remaining is spread through > 40 genes. This assumption is corroborated by analogous results obtained with ES-based studies analyzed. However, also, the peculiar clinical features of selected patients had a pivotal role in reaching a positive molecular diagnosis (i.e., late onset and presence of familial history).

In our cohort, *STUB1*, a gene originally described in SCAR16 and recently also associated with SCA48 [64], was found to be the most frequent disease-causing gene in HA [61,63], as also confirmed by others [95,96]. We cannot, however, exclude that the relative novelty of this gene might be a source of bias explaining its high rate in our study. *PRKCG*, a gene known to cause a rare form of ataxia (SCA14), was also found to be common [53], as was *SPG7*, which had a similar high frequency [97,98]. *PNPLA6*, a gene originally associated with HSP [47,99], was quite common, as too was *TMEM240* [67,100]. These findings suggest that it is worth testing sporadic HA patients for the rarer non-polyQ SCA forms.

Protein homeostasis and quality control, especially mitochondrial assembly and signaling, emerged as the biological pathways most frequently impaired in HA [101]; furthermore, a high prevalence of mutations in genes coding for ion channels or their subunits confirms recent findings indicating their high frequency in SCA [25,90]. Conversely, mutations in genes involved in signal transduction, cell development, DNA and RNA maintenance, metabolism of complex lipids, transport proteins, intracellular transport, and electron transport appear less common [4,101]. Other cellular processes such as those involving tRNA synthetases are seldom affected [8,29].

A sizeable proportion of the patients in our study harbored VOUS, and therefore had an uncertain molecular diagnosis. Like the patients with a defined genetic diagnosis, these cases harbored mostly (~75%) missense mutations, and this fact further highlights the need, in medical genetics, for robust functional tools for variant interpretation. In the case of VOUS, in silico predictions alone cannot suffice, and family studies often remain inconclusive. Therefore, efforts should be made to include functional analyses in the process of assessing and validating the putative pathogenic role of new variants. For instance, systematic use of simple in vivo models to predict the impact of new variants (e.g., complementation assays in yeast) could be a relatively fast and efficient method [21]. Our data also highlight the difficulties in providing certain genetic diagnoses, and therefore adequate counseling, to sporadic patients, especially in cases where it is not possible to investigate close family members, or there is limited access to clinical data. Future NGS studies in HA would certainly benefit from more appropriate sample/data collection, even more robust bioinformatic tools, and technical improvements facilitating phase attribution of variants (i.e., long-read sequencing [102]).

Notably, recent literature, including studies performed by our group, clearly suggest that the significance of variants should not be inferred from the clinical features of the index case and the known pattern of zygosity, because further heterogeneity and atypical phenotypes are emerging all the time, and the list of causative genes, both dominant and recessive, is growing rapidly [64]. Moreover, thanks to the availability of large, rapidly consultable genetic data repositories shared by multiple laboratories worldwide (such as the GENESIS 2.0 platform; https://www.tgp-foundation.org/, latest access 29 December 2020) [103], and the contribution of ataxia experts, it is also becoming possible to filter rare variants absent in public databases and avoid false interpretations [104].

Current computational methods predicting changes in protein stability seem to provide a reliable tool to confirm the deleterious effects of missense variants. On the other hand, these methods require a solved 3D protein structure, currently not available for several proteins, and often consisting of only a partial structure for most. Furthermore, these predictions do not take account of specific protein–protein interactions that are crucial in polygenic inheritance and in protein complexes assembly, probably explaining, for instance, why p. Val571Gly mutations in *AFG3L2* (inherited together with p. Ala510Val in *SPG7* in one patient) does not computationally sort any destabilizing effect on protein structure. However, the outcomes achieved combining in silico and 3D-modeling studies allowed us to speculate on the potential causative role of the other variants (i.e., those whose 3D protein structure was not available)

Interestingly, the SCAR genes considered to be frequent in the pre-NGS era (e.g., *ATM*, *SETX*, and *APTX*) were less common than expected in our cohort, or even absent (i.e., *SACS*). This could be related to the prevalently retrospective nature of our study, in which patients with peculiar phenotypes, such as those resembling ataxia-telangectasia, ataxia with oculomotor apraxia, and autosomal recessive spastic ataxia of Charlevoix-Saguenay (ARSACS), underwent direct Sanger sequencing prior to inclusion [105].

It is worth mentioning the translational value of our study. For instance, we identified a child harboring a de novo truncating mutation in *SLC2A1*, encoding the major glucose transporter in the brain [106], a critical finding that allowed the child to be promptly put on a ketogenic diet, which led to a significant clinical improvement (personal communication to FMS). Furthermore, our findings were helpful in identifying novel neuroimaging biomarkers in SCA48 [107] that could facilitate future diagnosis.

The TRP analyses were negative in two-thirds of our patients, as is commonly the case in all neurological disorders when using NGS applications [108]. Variants in noncoding parts of the genome (e.g., the intronic *RCF1* expansion) may account for unsolved cases, but functional tests remain a challenge. Furthermore, as recently observed in ataxia linked to both ultra-rare (i.e., *POLR3A*) and relatively more common (i.e., *SPG7*) genes [52,109], the presence of deep intronic mutations (not usually sought in TRP studies) might also explain these difficulties. The use of molecular karyotype analysis in sporadic cases might unveil de novo quantitative alterations not detectable by routine NGS applications. Pathogenic mutations misclassified as benign are presumably a common bias generating false-negative results in every NGS study. Indeed, systematic re-examinations of NGS data in the face of novel clinical insights, as well as more efficient combination of bioinformatic tools with system biology information, could increase the rate of positive diagnoses [88]. Non-genetic factors, such as epigenetic, post-transcriptional or environmental factors, might also play a role; the same applies to co-occurrence of variants in different genes that may exert a synergistic effect in the development of the disease. This latter phenomenon was found in our study for example (i.e., in the form of digenic mutations in *SPG7* and *AFG3L2*), and it has also been observed by others [95,110]. Intriguingly, recent studies indicate that the even more complex inheritance mechanisms of classical Mendelian disorders, such as multilocus inheritance, are emerging in inherited neurological disorders [111]. Against this background, it seems clear that the genetics of HA has new surprises in store for the future.

In summary, both our own experience and our literature analysis underline that a core set of a few dozen genes is the cause of most non-polyQ forms of HA, and highlight the existence of “more common”, “relatively rare”, and “ultra-rare” HA genes. Our experience suggests that TRPs are still a robust tool in clinical practice, and if combined with informative clinical data are worth adopting in large-scale genetic screenings.

## 4. Materials and Methods

### 4.1. Patient Recruitment

All samples were collected in centers belonging to ITASPAX (the Italian Spastic Paraplegia and Ataxia Network, coordinated by AF and FMS). The patient recruitment and biological sample collection stages of the study were performed during the six-year period 2015–2020. Individuals with acquired forms of ataxia were not included. A clinical diagnosis of genetically uncharacterized HA was the only inclusion criterion used, as the aim was to obtain a sample that reflected the routine clinical practice scenario. Therefore, patients were included regardless of their age at onset, their clinical features, and of the presence/absence of a family history of the disease and/or relatives available for segregation analysis.

### 4.2. DNA Extraction and Preliminary Analyses of Repeated Nucleotide Expansions

Genomic DNA was obtained using the MagPurix Blood DNA Extraction Kit 200 designed for the MagPurix DNA Extract (Zinexts, Zhonghe, Taiwan). Before undergoing massive parallel sequencing, all patients were tested for pathological trinucleotide expansions in SCA1, 2, 3, 6, 7, 8, 12, 17 using a TP-PCR-based method [112,113], and for the intronic GAA expansion in *FXN* using an established long-PCR technique [114]. Capillary sequencing for TP-PCR products was performed using a 3130xl Genetic Analyzer (Thermo Fisher Scientific, Waltham, MA, USA), and fragment analyses were performed using GeneMapper ID Software version 3.1 (Thermo Fisher Scientific).

### 4.3. Massive Parallel Sequencing and Data Analysis

A custom targeted resequencing panel encompassing 285 genes known to cause HA or more complex syndromes in which ataxia is a symptom was designed using SureDesign (Agilent Technologies, Santa Clara, CA, USA) (full list of genes available in Table S9). Fifty bases upstream and downstream of every coding exon were also covered, and designed probes were predicted to cover 99.5% of the whole region of interest. Library preparations were realized following the manufacturer’s instructions. Massive parallel sequencing was carried out using a NextSeq500 (Illumina, San Diego, CA, USA) sequencer.

Raw data alignment to the reference human genome sequence was carried out using SureCall (Agilent Technologies), while Ingenuity Variant Analysis (Qiagen, Venlo, The Netherlands) was used for the variant calling process. Single nucleotide variations and small insertions and deletions were selected using the following criteria: (i) quality score > 30; (ii) at least 30X of coverage (coverage was checked manually using Integrative Genomics Viewer, IGV, https://software.broadinstitute.org/software/igv/ whenever necessary, latest access 15 December 2020); (iii) MAF (minor allele frequency) < 1% in the ExAC (http://exac.broadinstitute.org/, latest access 15 December 2020), gnomAD (https://gnomad.broadinstitute.org/, latest access 15 December 2020), 1000 Genomes Project (https://www.internationalgenome.org/, latest access 15 December 2020), and dbSNP (https://www.ncbi.nlm.nih.gov/snp/, latest access 15 December 2020) databases; (iv) homozygous count < 3 in ExAC and gnomAD; (v) nonsynonymous variants in coding or splicing regions. Instead, synonymous, intronic, and other noncoding variants were considered only if they have already been described as pathogenic. SureCall and IGV were used for coverage analysis to detect large homozygous deletions in genes known to be prone to these rearrangements.

Variant classification was based on the American College of Medical Genetics and Genomics published guidelines [115]. To define the impact of missense mutations on protein function, we used an in silico pipeline encompassing 22 prediction tools including MutationTaster (http://www.mutationtaster.org/, latest access 22 December 2020), Mutation Assessor (http://mutationassessor.org/r3/, latest access 22 December 2020), FATHMM (http://fathmm.biocompute.org.uk/, latest access 22 December 2020), FATHMM-MKL (http://fathmm.biocompute.org.uk/fathmmMKL.html/, latest access 22 December 2020), FATHMM-XF (http://fathmm.biocompute.org.uk/fathmm-xf/, latest access 22 December 2020), LRT (https://varsome.com/, latest access 22 December 2020), Deogen2(https://varsome.com/, latest access 22 December 2020), Eigen (http://www.columbia.edu/~ii2135/eigen.html/, latest access 22 December 2020), Eigen PC (http://www.columbia.edu/~ii2135/eigen.html/, latest access 22 December 2020), SIFT (https://sift.bii.a-star.edu.sg/, latest access 22 December 2020), SIFT4G (https://sift.bii.a-star.edu.sg/sift4g/, latest access 22 December 2020), Provean (http://provean.jcvi.org/index.php/, latest access 22 December 2020), MVP (https://varsome.com/, latest access 22 December 2020), Revel (https://varsome.com/, latest access 22 December 2020), Primate AI (https://varsome.com/, latest access 22 December 2020), MetaSVM (https://varsome.com/, latest access 22 December 2020), MetalR (https://varsome.com/, latest access 22 December 2020), GERP (https://varsome.com/, latest access 22 December 2020), PolyPhen-2 HumDir (http://genetics.bwh.harvard.edu/pph2/, latest access 22 December 2020), PolyPhen-2 HumVar (http://genetics.bwh.harvard.edu/pph2/, latest access 22 December 2020), UMD Predictor (http://umd-predictor.eu/, latest access 22 December 2020), and CADD (https://cadd.gs.washington.edu/, latest access 22 December 2020). Splicing variants and synonymous variants close to splicing sites were also tested using Human Splicing Finder 3.1 (http://www.umd.be/HSF/, latest access 22 December 2020) and NNSPLICE 0.9 (http://www.fruitfly.org/seq_tools/splice.html/, latest access 22 December 2020). Thus, variants were further filtered using highly stringent criteria, to identify those with a CADD score > 20 and for which the majority (more than half) of other algorithms suggested a damaging effect.

Filtered variants were also explored in the PREPARE-Ataxia network genomes (https://www.prepare-ataxia.com/, latest access 29 December 2020). This was done in the GENESIS 2.0 platform (https://www.tgp-foundation.org/, latest access 29 December 2020), an affordable genome-scale analysis and data management solution for medical research containing genomic data of over 12,000 individuals with rare neurological diseases and both affected and healthy relatives, to find a match with other affected individuals, or to exclude variants present in healthy individuals in the case of dominant genes, or genes characterized by high frequency. Use of the Human Gene Mutation Database was not deemed mandatory in order to classify variants as disease causing.

### 4.4. Sanger Sequencing

Regions containing selected variants of interest were amplified by PCR. PCR products were purified with ExoSAP-IT™ PCR Product Cleanup Reagent (Thermo Fisher Scientific) and sequenced using the BigDye™ Terminator v3.1 Cycle Sequencing Kit (Thermo Fisher Scientific) in a 3500xL Genetic Analyzer (Thermo Fisher Scientific). Electropherograms were analyzed using SeqScape™ Software v3.0 (Thermo Fisher Scientific).

### 4.5. Multiplex Ligation-Dependent Probe Amplification (MLPA) Analysis

In order to find second mutations, MLPA (MRC-Holland, Amsterdam, The Netherlands) was performed to identify deletions/duplications in patients harboring a single mutation in a frequent recessive gene. We used Salsa kits P213 for *SPG7*, P441 for *SACS*, and P163 for *WFS1*. Capillary sequencing for MLPA products was performed using 3130xl Genetic Analyzer (Thermo Fisher Scientific), while Coffalyser software (MRC-Holland) was employed to analyze MLPA results.

### 4.6. Computational Analysis of Protein Stability

To predict protein stability changes upon mutation, in terms of variation in folding free energy (ΔΔG) between wild type and mutant structures, we employed five different computational methods: DynaMut [116], ENCoM [117], mCSM [118], SDM [119], and DUET [120]. This analysis was performed on those variants that were novel and whose 3D structure was partially or completely solved and available in Protein Data Bank (https://www.rcsb.org/, latest access 25 May 2021). DynaMut web server (http://biosig.unimelb.edu.au/dynamut/, latest access 25 May 2021) was used to perform all predictions, and to generate images of interatomic interactions.

### 4.7. Literature Revision

To analyze the state-of-the-art in HA gene testing, we looked for relevant literature published since 2013, querying PubMed and Google Scholar with pertinent keywords (latest access 4 January 2021). To allow a reliable comparison between our study and those reported in the literature, we specifically selected cohort studies involving small, medium and large cohorts of patients with a clinical diagnosis of HA but no diagnosis on common genetic tests, regardless of other specific features.

## 5. Conclusions

The application of a TRP in 377 patients with a clinical diagnosis of HA yielded a molecular diagnosis in one out of three patients, twice the diagnostic yield reported in similar published studies employing TRPs, and in line with the results of ES.

Our study allows a series of considerations. First, it seems that genomic results should be considered dynamic, not static, data, and we strongly encourage their sharing through appropriate platforms, especially in the case of “ultra-rare” HA genes. Second, both our experience and data published by others confirm that NGS is a far-from-perfect tool. Even though we broadened the genetic and clinical spectrum of HA, two-thirds of our cases remained unsolved, confirming the results of previous studies. It is tempting to speculate that we might have reached a sort of “plateau” in our ability to find answers in the coding parts of the genome; this clearly makes use of WGS a much-needed next step in medical genetics. Other boundaries include zygosity and formal clinical classifications, with the latter now being almost obsolete. Moreover, consideration should also be given to the possibility of identifying additional minor variants acting as potential modifiers, or even cases of complex multilocus inheritance. Similarly, a more correct integration of genetic data with results of other omics approaches may unveil non-genetic causes of HA. Overcoming all these challenges will probably move us towards an even higher diagnostic rate, by allowing us to solve “cold cases” in several neurological conditions, including HA.

## Figures and Tables

**Table 1:** Summary of NGS results in positive cases harboring pathogenic and likely pathogenic variants.

Index Case	Gender, Age	Transmission/Onset	Gene (Ref_Seq)	cDNA Variant	Protein Variant	Zygosity	Type	Reference
Pt1	F, 36	AR/Early	*AARS2* (NM_020745.4)	c.385A > C	p.Thr129Pro	Het	Missense	[8]
				c.446G > A	p.Cys149Tyr	Het	Missense	
Pt2	M, 37	SP/Early	*ABCD1* (NM_000033.4)	c.1661G > A	p.Arg554His	Hem	Missense	[9]
Pt3	M, 50	SP/Late	*ABCD1* (NM_000033.4)	c.2087A > T	p.Lys696Met	Hem	Missense	This study
Pt4	M, 11	SP/Childhood	*AFG3L2* (NM_006796.3)	c.634dupG	p.Val212GlyfsTer4	Het	Frameshift	[10]
				c.2167G > A	p.Val723Met	Het	Missense	[11]
Pt5	F, 26	AD/Childhood	*AFG3L2* (NM_006796.3)	c.2105G > A	p.Arg702Gln	Het	Missense	[12]
Pt6	M, 65	SP/Early	*AFG3L2* (NM_006796.3)	c.1712T > G	p.Val571Gly	Het	Missense	This study
			*SPG7 * (NM_003119.4)	c.1529C > T	p.Ala510Val	Het	Missense	[13]
Pt7	M, 58	AR/Early	*ANO10* (NM_018075.5)	c.289delA	p.Met97Ter	Het	Nonsense	This study
				c.1009T > G	p.Phe337Val	Het	Missense	[14]
Pt8	F, 52	SP/Late	*ANO10* (NM_018075.5)	c.289delA	p.Met97Ter	Hom	Nonsense	This study
Pt9	F, 43	SP/Early	*ANO10* (NM_018075.5)	c.206T > A	p.Leu69Ter	Hom	Nonsense	This study
Pt10	M, 31	SP/Early	*APTX* (NM_001195248.2)	c.544-1G > C	NA	Het	Splicing	This study
				c.668T > C	p.Leu223Pro	Het	Missense	[15]
Pt11	M, 2	SP/Childhood	*ATM * (NM_000051.3)	c.2152dupT	p.Cys718LeufsTer20	Het	Frameshift	This study
				c.2929T > C	p.Cys977Arg	Het	Missense	
P12	M, 7	SP/Childhood	*ATM* (NM_000051.3)	c.3802delG	p.Val1268Ter	Het	Nonsense	[16]
				c.3894dupT	p.Ala1299CysfsTer3	Het	Frameshift	[17]
Pt13	M, 50	SP/Childhood	*ATM * (NM_000051.3)	c.6650_6657delTTAGTTTT	p.Phe2217SerfsTer29	Het	Frameshift	This study
				c.8147T > C	p.Val2716Ala	Het	Missense	[18]
Pt14	M, 77	AR/Early	*ATP13A2* (NM_022089.4)	c.1205C > T	p.Thr402Met	Hom	Missense	[19]
Pt15	F, 12	SP/Childhood	*CACNA1A* (NM_001127222.2)	c.4897G > A	p.Asp1633Asn	Het	Missense	This study
Pt16	F, 43	SP/Early	*CACNA1A* (NM_001127222.2)	c.3310_3315dupGGCCCC	p.Gly1104_Pro1105dup	Het	Inframe dup	This study
Pt17	F, 18	SP/Childhood	*CACNA1A* (NM_001127222.2)	c.4927G > A	p.Asp1643Asn	Het	Missense	This study
Pt18	F, 49	SP/Late	*CACNA1A* (NM_001127222.2)	c.4466T > C	p.Ile1489Thr	Het	Missense	This study
Pt19	F, 62	SP/Late	*CACNA1G* (NM_018896.5)	c 481A > T	p.Ile161Phe	Het	Missense	This study
Pt20	F, 59	AD/Early	*CACNA1G* (NM_018896.5)	c.5144G > A	p.Arg1715His	Het	Missense	[20]
Pt21	M, 48	SP/Late	*CACNA1G* (NM_018896.5)	c.5960_5961delCGinsAC	p.Thr1987Asn	Het	Missense	This study
Pt22	M, 55	SP/Late	*CACNA1G* (NM_018896.5)	c.3835G > A	p.Asp1279Asn	Het	Missense	This study
Pt23	M, 5	SP/Childhood	*COQ4 * (NM_016035.5)	c.577C > T	p.Pro193Ser	Het	Missense	[21]
				c.718C > T	p.Arg240Cys	Het	Missense	[22]
Pt24	F, 20	SP/Early	*COQ4* (NM_016035.5)	c.284G > A	p.Gly95Asp	Het	Missense	[21]
				c.305G > A	p.Arg102His	Het	Missense	
Pt25	F, 10	SP/Childhood	*COQ8A* (NM_020247.5)	c.589-3C > G	NA	Het	Splicing	[23]
				c.1844G > A	p.Gly615Asp	Het	Missense	
Pt26	M, 55	SP/Early	*COQ8A* (NM_020247.5)	c.1042C > T	p.Arg348Ter	Hom	Nonsense	[24]
Pt27	M, 65	SP/Late	*COQ8A* (NM_020247.5)	c.127delC	p.Leu43CysfsTer166	Het	Frameshift	[25]
				c.1376T > C	p.Leu459Pro	Het	Missense	This study
Pt28	F, 61	SP/Late	*DNMT1* (NM_001130823.3)	c.1709C > T	p.Ala570Val	Het	Missense	[26]
Pt29	M, 53	SP/Late	*ERCC4* (NM_005236.3)	c.1730dupA	p.Tyr577Ter	Het	Nonsense	This study
				c.2248C > T	p.Arg750Cys	Het	Missense	
Pt30	M, 27	AR/Childhood	*EXOSC3* (NM_016042.4)	c.395A > C	p.Asp132Ala	Hom	Missense	[27]
Pt31	F, 48	SP/Late	*GJC2* (NM_020435.4)	c.219_220delCC	p.Leu74ValfsTer33	Het	Frameshift	This study
				c.254T > C	p.Val85Ala	Het	Missense	
Pt32	M, 11	SP/Childhood	*HARS1* (NM_002109.6)	c.616G > T	p.Asp206Tyr	Het	Missense	[28]
				c.730delG	p.Val244CysfsTer6	Het	Frameshift	
Pt33	F, 39	AR/Childhood	*HARS1* (NM_002109.6)	c.910_912dupTTG	p.Leu305dup	Het	In-frame dup	[28]
				c.1393A > C	p.Ile465Leu	Het	Missense	
Pt34	M, 32	SP/Early	*HSD17B4* (NM_001199291.3)	c.727G > T	p.Val243Leu	Het	Missense	[29]
				c.2191C > T	p.Gln731Ter	Het	Nonsense	This study
Pt35	M, 6	SP/Childhood	*ITPR1* (NM_001168272.1)	c.722G > A	p.Arg241Lys	Het	Missense	[30]
Pt36	M, 31	SP/Childhood	*ITPR1* (NM_001168272.1)	c.7748T > C	p.Ile2583Thr	Het	Missense	[31]
Pt37	M, 2	SP/Childhood	*ITPR1* (NM_001168272.1)	c.805C > T	p.Arg269Trp	Het	Missense	[30]
Pt38	M, 74	SP/Late	*ITPR1* (NM_001099952.3)	c.2816G > A	p.Gly939Glu	Het	Missense	This study
Pt39	M, 24	SP/Early	*KCNA2* (NM_004974.4)	c.890G > A	p.Arg297Gln	Het	Missense	[32]
Pt40	M, 19	SP/Early	*KCNA2* (NM_004974.4)	c.881G > A	p.Arg294His	Het	Missense	[33]
Pt41	F, 63	AD/Childhood	*KCNC3* (NM_004977.3)	c.1268G > A	p.Arg423His	Het	Missense	[34]
Pt42	M, 50	SP/Late	*KCND3* (NM_004980.4)	c.1646G > A	p.Arg549His	Het	Missense	[35]
Pt43	M, 80	AD/Early	*KCND3* (NM_004980.4)	c.680_682delTCT	p.Phe227del	Het	In-frame del	[36]
Pt44	M, 28	SP/Childhood	*KCND3* (NM_004980.4)	c.611C > T	p.Thr204Met	Het	Missense	This study
Pt45	M, 42	SP/Childhood	*KIF1A* (NM_001244008.1)	c.609_610delGAinsAAAAG	p.Arg203_Thr204delins	Het	In-frame indel	This study
					GlyLysAla			
Pt46	F, 57	SP/Late	*KIF1A* (NM_001244008.1)	c.32G > A	p.Arg11Gln	Het	Missense	[37]
Pt47	M, 29	SP/Childhood	*KIF1C * (NM_006612.6)	c.765delC	p.Asp256ThrfsTer10	Hom	Frameshift	This study
Pt48	M, 7	SP/Childhood	*LAMA1* (NM_005559.4)	c.184C > T	p.Arg62Ter	Het	Nonsense	This study
				c.1404_1405delAG	p.Gly469AlafsTer5	Het	Frameshift	[38]
Pt49	F, 32	SP/Early	*MFN2* (NM_001127660.1)	c.1987C > T	p.Arg663Cys	Het	Missense	[39]
Pt50	F, 83	AD/Late	*MMACHC* (NM_015506.3)	c.271dupA	p.Arg91LysfsTer14	Het	Frameshift	[40]
				c.472T > C	p.Phe158Leu	Het	Missense	[41]
Pt51	F, 56	SP/Late	*MME * (NM_007288.3)	c.2154G > T	p.Arg718Ser	Het	Missense	This study
Pt52	M, 39	SP/Early	*OPA1 * (NM_130837.2)	c.885C > G	p.Asn295Lys	Het	Missense	This study
Pt53	M, 65	SP/Late	*OPA1 * (NM_130837.2)	c.2873_2876delTTAG	p.Val958GlyfsTer3	Het	Frameshift	[42]
Pt54	F, 5	SP/Childhood	*PLA2G6* (NM_003560.4)	c.1111G > A	p.Val371Met	Het	Missense	[43]
				c.1703T > C	p.Phe568Ser	Het	Missense	This study
Pt55	M, 33	AR/Childhood	*PMM2* (NM_000303.3)	c.323C > T	p.Ala108Val	Het	Missense	[44]
				c.422G > A	p.Arg141His	Het	Missense	
Pt56	F, 42	AR/Early	*PNPLA6* (NM_001166111.2)	c.1880C > T	p.Ala627Val	Hom	Missense	This study
Pt57	F, 57	SP/Late	*PNPLA6* (NM_001166111.2)	c.2264A > C	p.Gln755Pro	Het	Missense	This study
				c.3388C > T	p.His1130Tyr	Het	Missense	
Pt58	M, 56	SP/Late	*PNPLA6* (NM_001166111.2)	c.3023A > G	p.Asp1008Gly	Het	Missense	This study
				c.4075C > T	p.Arg1359Trp	Het	Missense	[45]
Pt59	F, 64	AR/Late	*PNPLA6* (NM_001166111.2)	c.3385G > A	p.Gly1129Arg	Hom	Missense	[46]
Pt60	M, 33	SP/Early	*PNPLA6* (NM_001166111.2)	c.3365C > T	p.Pro1122Leu	Het	Missense	[47]
				c.4081C > T	p.Arg1361Ter	Het	Nonsense	[48]
Pt61	F, 70	SP/Late	*POLG* (NM_001126131.2)	c.752C > T	p.Thr251Ile	Het	Missense	[49]
				c.1760C > T	p.Pro587Leu	Het	Missense	[50]
				c.2243G > C	p.Trp748Ser	Het	Missense	[51]
Pt62	F, 65	AR/Late	*POLR3A* (NM_007055.4)	c.1909 + 22G > A	NA	Het	Splicing	[52]
				c.4073G > A	p.Gly1358Glu	Het	Missense	This study
Pt63	M, 35	SP/Childhood	*POLR3A* (NM_007055.4)	c.1909 + 22G > A	NA	Het	Splicing	[52]
				Deletion ex. 14-18	NA	Het	Large deletion	This study
Pt64	M, 65	AR/Late	*POLR3A* (NM_007055.4)	c.1909 + 22G > A	NA	Het	Splicing	[52]
				c.3839dupT	p.Met1280IlefsTer20	Het	Frameshift	This study
Pt65	M, 69	AD/Late	*PRKCG* (NM_002739.5)	c.230G > A	p.Cys77Tyr	Het	Missense	[53]
Pt66	M, 50	AD/Late	*PRKCG* (NM_002739.5)	c.358C > T	p.Leu120Phe	Het	Missense	[53]
Pt67	F, 63	SP/Late	*PRKCG* (NM_002739.5)	c.1928T > G	p.Phe643Cys	Het	Missense	[53]
Pt68	F, 47	SP/Late	*PRKCG* (NM_002739.5)	c.1381G > A	p.Ala461Thr	Het	Missense	[53]
Pt69	M, 40	AD/Childhood	*PRKCG* (NM_002739.5)	c.466G > A	p.Glu156Lys	Het	Missense	[53]
Pt70	M, 7	SP/Childhood	*PRKCG* (NM_002739.5)	c.1308C > G	p.Tyr436Ter	Het	Nonsense	[53]
Pt71	F, 35	SP/Early	*PRKCG* (NM_002739.5)	c.413T > A	p.Val138Glu	Het	Missense	[54]
Pt72	M, 41	SP/Early	*PRKCG* (NM_002739.5)	c.380A > C	p.Gln127Pro	Het	Missense	[55]
Pt73	F, 49	SP/Late	*PRKCG* (NM_002739.5)	c.230G > A	p.Cys77Tyr	Het	Missense	[53]
Pt74	F, 14	SP/Childhood	*PRKCG* (NM_002739.5)	c.419G > A	p.Arg140Gln	Het	Missense	[53]
Pt75	F, 66	AD/Late	*PRNP* (NM_001080123.3)	c.305C > T	p.Pro102Leu	Het	Missense	This study
Pt76	M, 45	SP/Late	*PSEN1* (NM_000021.4)	c.300dupT	p.Lys101Ter	Het	Nonsense	This study
Pt77	F, 10	SP/Childhood	*RARS2* (NM_020320.5)	c.517G > A	p.Asp173Asn	Het	Missense	This study
				c.1037C > T	p.Thr346Ile	Het	Missense	
Pt78	F, 27	SP/Childhood	*RNF170* (NM_030954.4)	c.566T > G	p.Phe189Cys	Het	Missense	This study
Pt79	F, 45	SP/Early	*RNF216* (NM_207111.4)	c.1849A > G	p.Met617Val	Het	Missense	[56]
				c.2061 + 3A > G	NA	Het	Splicing	
Pt80	M, 52	SP/Childhood	*SETX * (NM_015046.7)	c.7292dupA	p.Asn2431LysfsTer19	Hom	Frameshift	This study
Pt81	M, 87	SP/Late	*SETX* (NM_015046.7)	c.5591A > C	p.Gln1864Pro	Het	Missense	This study
Pt82	F, 5	SP/Childhood	*SLC2A1* (NM_006516.3)	c.136C > T	p.Gln46Ter	Het	Nonsense	[57]
Pt83	F, 15	SP/Childhood	*SLC2A1* (NM_006516.3)	c.985G > A	p.Glu329Lys	Het	Missense	This study
Pt84	M, 6	SP/Childhood	*SLC9A6* (NM_001177651)	Deletion ex. 4-7	NA	Hem	Large deletion	This study
Pt85	F, 71	SP/Late	*SPG7 * (NM_003119.4)	c.679C > T	p.Arg227Ter	Het	Nonsense	[58]
				c.1231G > A	p.Asp411Asn	Het	Missense	
Pt86	F, 57	SP/Early	*SPG7 * (NM_003119.4)	c.1529C > T	p.Ala510Val	Het	Missense	[13]
				c.1940C > A	p.Ala647Glu	Het	Missense	This study
Pt87	M, 72	AR/Late	*SPG7 * (NM_003119.4)	c.1529C > T	p.Ala510Val	Hom	Missense	[13]
Pt88	M, 65	AR/Late	*SPG7* (NM_003119.4)	c.1529C > T	p.Ala510Val	Hom	Missense	[13]
Pt89	M, 25	AR/Early	*SPG7* (NM_003119.4)	Deletion ex. 2	NA	Hom	Large deletion	This study
Pt90	M, 69	AR/Early	*SPG7 * (NM_003119.4)	c.73_80delCCAGGCCC	p.Pro25GlyfsTer46	Het	Frameshift	This study
				c.1940C > A	p.Ala647Glu	Het	Missense	
Pt91	M, 46	SP/Early	*SPG7 * (NM_003119.4)	Deletion ex. 2	NA	Hom	Large deletion	This study
Pt92	F, 63	AR/Late	*SPG7* (NM_003119.4)	c.1529C > T	p.Ala510Val	Het	Missense	[13]
				c.1972G > A	p.Ala658Thr	Het	Missense	[59]
Pt93	F, 36	SP/Childhood	*SPTAN1* (NM_001363759.2)	c.4870C > T	p.Arg1624Cys	Het	Missense	This study
Pt94	M, 60	SP/Late	*SPTBN2* (NM_006946.3)	c.5066G > A	p.Arg1689His	Het	Missense	This study
Pt95	M, 26	SP/Early	*SPTBN2* (NM_006946.3)	c.1438C > T	p.Arg480Trp	Het	Missense	[60]
Pt96	M, 54	AR/Late	*SPTBN2* (NM_006946.3)	c.157 + 1G > A	NA	Het	Splicing	This study
				c.1843C > T	p.Arg615Trp	Het	Missense	
Pt97	M, 63	AD/Late	*STUB1* (NM_005861.4)	c.97G > A	p.Gly33Ser	Het	Missense	[61]
Pt98	M, 52	AD/Early	*STUB1* (NM_005861.4)	c.689_692delACCT	p.Tyr230CysfsTer9	Het	Frameshift	[62]
Pt99	M, 61	AD/Late	*STUB1* (NM_005861.4)	c.682C > T	p.Pro228Ser	Het	Missense	[61]
Pt100	F, 53	SP/Early	*STUB1* (NM_005861.4)	c.199G > A	p.Ala67Thr	Het	Missense	[63]
Pt101	F, 48	AD/Late	*STUB1* (NM_005861.4)	c.673C > T	p.Arg225Ter	Het	Nonsense	[63]
Pt102	F, 55	SP/Late	*STUB1* (NM_005861.4)	c.721C > T	p.Arg241Trp	Het	Missense	[63]
Pt103	M, 48	SP/Late	*STUB1* (NM_005861.4)	c.433A > C	p.Lys145Gln	Het	Missense	[62]
Pt104	M, 52	AD/Late	*STUB1* (NM_005861.4)	c.170C > T	p.Pro57Leu	Het	Missense	[63]
Pt105	F, 67	AD/Late	*STUB1* (NM_005861.4)	c.818_819dupGC	p.Pro274AlafsTer3	Het	Frameshift	[63]
Pt106	F, 70	AD/Late	*STUB1* (NM_005861.4)	c.791_792delTG	p.Val264GlyfsTer4	Het	Frameshift	[63]
Pt107	F, 60	SP/Early	*STUB1* (NM_005861.4)	c.823_824delCT	p.Leu275AspfsTer16	Het	Frameshift	[64]
Pt108	F, NA	SP/NA	*STXBP1* (NM_003165.5)	c.874C > T	p.Arg292Cys	Het	Missense	[65]
Pt109	F, 9	SP/Childhood	*STXBP1* (NM_003165.5)	c.434A > G	p.Tyr145Cys	Het	Missense	This study
Pt110	F, 48	SP/Late	*STXBP1* (NM_003165.5)	c.298C > T	p.Arg100Trp	Het	Missense	This study
Pt111	M, 32	SP/Early	*SYNE1* (NM_182961.4)	c.15049C > T	p.Gln5017Ter	Hom	Nonsense	This study
Pt112	M, 61	SP/Late	*SYNE1* (NM_182961.4)	c.6724-1G > A	NA	Het	Splicing	This study
				c.7085dupA	p.Asn2362LysfsTer4	Het	Frameshift	
Pt113	F, 36	AR/Early	*SYNE1* (NM_182961.4)	c.4609C > T	p.Arg1537Ter	Hom	Nonsense	This study
Pt114	F, 38	AR/Early	*SYNE1* (NM_182961.4)	c.3130C > T	p.Arg1044Ter	Het	Nonsense	This study
				c.7911G > A	p.Trp2637Ter	Het	Nonsense	
Pt115	M, 3	SP/Childhood	*SYNE2* (NM_182914.2)	c.2970C > A	p.Tyr990Ter	Het	Nonsense	This study
Pt116	M, 42	SP/Early	*TMEM240* (NM_001114748.1)	c.509C > T	p.Pro170Leu	Het	Missense	[66]
Pt117	M, 46	AD/Late	*TMEM240* (NM_001114748.1)	c.509C > T	p.Pro170Leu	Het	Missense	[66]
Pt118	M, 7	SP/Childhood	*TMEM240* (NM_001114748.1)	c.196G > A	p.Gly66Arg	Het	Missense	[67]
Pt119	F, 64	SP/Early	*TMEM240* (NM_001114748.1)	c.419T > A	p.Leu140Gln	Het	Missense	This study
Pt120	M, 12	SP/Childhood	*TMEM240* (NM_001114748.1)	c.239C > T	p.Thr80Met	Het	Missense	[66]
Pt121	M, 6	SP/Childhood	*TPP1* (NM_000391.4)	c.225A > G	p.Gln75=	Hom	Splicing	[68]
Pt122	F, 64	SP/Late	*TRPC3* (NM_001130698.2)	c.1419delT	p.Val474CysfsTer29	Het	Frameshift	This study
Pt123	F, 73	SP/Late	*TTBK2* (NM_173500.4)	c.239T > A	p.Phe80Tyr	Het	Missense	This study
Pt124	F, 74	SP/Late	*TTPA * (NM_000370.3)	c.553-1G > T	NA	Hom	Splicing	This study
Pt125	M, 53	SP/Late	*WFS1* (NM_006005.3)	c.1291G > C	p.Glu431Gln	Het	Missense	This study
				c.1523A > G	p.Tyr508Cys	Het	Missense	[69]

**Table 2:** Ataxia-cohort studies published so far in literature.

Study	NGS Application	Index Cases	Main Cohort Feature(s)	Diagnostic Yield (%)
Nemeth et al., 2013 [6]	TRP (118 genes)	50	Heterogeneous	16
Ohba et al., 2013 [70]	ES	23	Childhood onset, sporadic	39
Sawyer et al., 2014 [71]	ES	28	Pediatric onset	39
Fogel et al., 2014 [72]	ES	76	Heterogeneous	21
Pyle et al., 2015 [73]	ES	22	Heterogeneous	41
Keogh et al., 2015 [74]	ES	12	Late onset	33
Mallaret et al., 2016 [75]	TRP (57 genes)	145	Onset < 60 years	16
van de Warrenburg et al., 2016 [76]	ES	28	Heterogeneous	32
Marelli et al., 2016 [77]	ES	33	Onset < 50 years	42
Kuperberg et al., 2016 [78]	ES	21	Pediatric onset	57
Hadjivassiliou et al., 2016 [79]	TRP (57 genes)	146	Progressive ataxia	24
Coutelier et al., 2017 [25]	TRP (65 genes)	412	Dominant inheritance	11
Iqbal et al., 2017 [80]	TRP (159 genes)	58	Heterogeneous	14
Nibbeling et al., 2017 [81]	ES	20	Dominant inheritance	35
Nibbeling et al., 2017 [81]	TRP (42 genes)	96	Dominant inheritance	15
Coutelier et al., 2018 [59]	ES	319	Heterogeneous	28
Montaut et al., 2018 [82]	ES	23	Heterogeneous	43
Dong et al., 2019 [83]	TRP (56 genes) + mDNA	33	Heterogeneous	15
Kang et al., 2019 [84]	TRP (46 and 98 genes)	32	Heterogeneous	25
Shakya et al., 2019 [85]	ES	16	Early onset	56
Shakya et al., 2019 [85]	TRP (41 genes)	82	Early onset	17
Sun et al., 2019 [86]	ES	170	Heterogeneous	52
Arslan et al., 2020 [87]	TRP (111 genes)	84	Pediatric onset	25
Ngo et al., 2020 [88]	ES	184	Heterogeneous	24
Mutlu-Albayrak et al., 2020 [89]	TRP (13 genes)	40	Consanguinity, childhood	82
			onset, recessive inheritance	
Gauquelin et al., 2020 [90]	ES	66	Heterogeneous	53
Ignatius et al., 2020 [91]	ES	50	Onset < 5 years	40
Kim et al., 2020 [92]	ES	68	Heterogeneous	26
Bogdanova-Mihaylova et al., 2021 [93]	ES	20	Progressive ataxia	20
Bogdanova-Mihaylova et al., 2021 [93]	TRP (87 genes) *	84	Progressive ataxia	36

* Average of several different panels employed.

## Data Availability

All variants described can be found in the ClinVar genetic repository (https://www.ncbi.nlm.nih.gov/clinvar/, latest access 27 July 2021; Submission IDs: SUB9291375 and SUB10090376, release is held until published).

## Supplementary Materials

The following are available online at www.mdpi.com/xxx/s1/, Table S1: In silico predictions, population frequencies, and ACMG classification of pathogenic and likely pathogenic variants in positive cases, Table S2: Protein domain localization analysis of novel missense variants of pathogenic significance, Table S3: Computational analysis of protein stability changes Table S4: Genetic features of patients with an uncertain molecular diagnosis, Table S5: Disease-causing genes in each study analyzed in the literature, Table S6: Types of pathogenic mutations identified in all the studies analyzed, Table S7: Frequency of all disease-causing genes described in the literature, Table S8: Efficiency of our TRP applied to literature data, Table S9: Full list of genes available in the TRP employed in this study, Figure S1: Conservation analysis through species of novel variants of pathogenic significance identified in this study, Figure S2: Interatomic interactions changes of missense variants computationally investigated. Wild-type and mutant residues are colored in light-green.

## References

[1] Jayadev S., Bird T.D. (2013). Hereditary ataxias: Overview. Genet. Med..

[2] Synofzik M., Schüle R. (2017). Overcoming the divide between ataxias and spastic paraplegias: Shared phenotypes, genes, and pathways. Mov. Disord..

[3] Ruano L., Silva M.C., Coutinho P. (2014). The global epidemiology of hereditary ataxia and spastic paraplegia: A systematic review of prevalence studies. Neuroepidemiology.

[4] Klockgether T., Mariotti C., Paulson H.L. (2019). Spinocerebellar ataxia. Nat. Rev. Dis. Prim..

[5] Galatolo D., Tessa A., Filla A., Santorelli F.M. (2018). Clinical application of next generation sequencing in hereditary spinocerebellar ataxia: Increasing the diagnostic yield and broadening the ataxia-spasticity spectrum. A retrospective analysis. Neurogenetics.

[6] Németh A.H., Kwasniewska A.C., Lise S., Parolin Schnekenberg R., Becker E.B.E., Bera K.D., Shanks M.E., Gregory L., Buck D., Zameel Cader M. (2013). Next generation sequencing for molecular diagnosis of neurological disorders using ataxias as a model. Brain.

[7] De Michele G., Galatolo D., Barghigiani M., Dello Iacovo D., Trovato R., Tessa A., Salvatore E., Filla A., De Michele G., Santorelli F.M. (2020). Spinocerebellar ataxia type 48: Last but not least. Neurol. Sci..

[8] De Michele G., Galatolo D., Lieto M., Maione L., Cocozza S., Santorelli F.M., Filla A. (2020). New *AARS2* mutations in two siblings with tremor, downbeat nystagmus, and primary amenorrhea: A benign phenotype without leukoencephalopathy. Mov. Disord. Clin. Pract..

[9] Korenke G.C., Roth C., Krasemann E., Hüfner M., Hunneman D.H., Hanefeld F. (1997). Variability of endocrinological dysfunction in 55 patients with X- linked adrenoleucodystrophy: Clinical, laboratory and genetic findings. Eur. J. Endocrinol..

[10] Tunc S., Dulovic-Mahlow M., Baumann H., Baaske M.K., Jahn M., Junker J., Münchau A., Brüggemann N., Lohmann K. (2019). Spinocerebellar ataxia type 28—Phenotypic and molecular characterization of a family with heterozygous and compound-heterozygous mutations in *AFG3L2*. Cerebellum.

[11] Dosi C., Galatolo D., Rubegni A., Doccini S., Pasquariello R., Nesti C., Sicca F., Barghigiani M., Battini R., Tessa A. (2020). Expanding the clinical and genetic heterogeneity of SPAX5. Ann. Clin. Transl. Neurol..

[12] Di Bella D., Lazzaro F., Brusco A., Plumari M., Battaglia G., Pastore A., Finardi A., Cagnoli C., Tempia F., Frontali M. (2010). Mutations in the mitochondrial protease gene *AFG3L2* cause dominant hereditary ataxia SCA28. Nat. Genet..

[13] Brugman F., Scheffer H., Wokke J.H.J., Nillesen W.M., De Visser M., Aronica E., Veldink J.H., Van Den Berg L.H. (2008). Paraplegin mutations in sporadic adult-onset upper motor neuron syndromes. Neurology.

[14] Renaud M., Anheim M., Kamsteeg E.J., Mallaret M., Mochel F., Vermeer S., Drouot N., Pouget J., Redin C., Salort-Campana E. (2014). Autosomal recessive cerebellar ataxia type 3 due to *ANO10* mutations: Delineation and genotype-phenotype correlation study. JAMA Neurol..

[15] Criscuolo C., Mancini P., Menchise V., Saccà F., De Michele G., Banfi S., Filla A. (2005). Very late onset in ataxia oculomotor apraxia type I. Ann. Neurol..

[16] McConville C.M., Stankovic T., Byrd P.J., McGuire G.M., Yao Q.Y., Lennox G.G., Taylor A.M.R. (1996). Mutations associated with variant phenotypes in ataxia-telangiectasia. Am. J. Hum. Genet..

[17] Teraoka S.N., Telatar M., Becker-catania S., Liang T., Suna O., Tolun A., Chessa L., Sanal O., Bernatowska E., Gatti R.A. (1999). Splicing defects in the ataxia-telangiectasia gene, *ATM*: Underlying mutations and consequences. Am. J. Hum. Genet..

[18] Scott S.P., Bendix R., Chen P., Clark R., Dörk T., Lavin M.F. (2002). Missense mutations but not allelic variants alter the function of *ATM* by dominant interference in patients with breast cancer. Proc. Natl. Acad. Sci. USA.

[19] De Michele G., Galatolo D., Lieto M., Fico T., Saccà F., Santorelli F.M., Filla A. (2020). Ataxia-myoclonus syndrome due to a novel homozygous *ATP13A2* mutation. Park. Relat. Disord..

[20] Coutelier M., Blesneac I., Monteil A., Monin M., Ando K., Mundwiller E., Brusco A., Le Ber I., Anheim M., Castrioto A. (2015). A recurrent mutation in *CACNA1G* alters Cav3.1 T-type calcium-channel conduction and causes autosomal-dominant cerebellar ataxia. Am. J. Hum. Genet..

[21] Mero S., Salviati L., Leuzzi V., Rubegni A., Calderan C., Nardecchia F., Galatolo D., Desbats M.A., Naef V., Gemignani F. (2021). New pathogenic variants in *COQ4* cause ataxia and neurodevelopmental disorder without detectable CoQ10 deficiency in muscle or skin fibroblasts. J. Neurol..

[22] Brea-Calvo G., Haack T.B., Karall D., Ohtake A., Invernizzi F., Carrozzo R., Kremer L., Dusi S., Fauth C., Scholl-Burgi S. (2015). *COQ4* mutations cause a broad spectrum of mitochondrial disorders associated with CoQ10 deficiency. Am. J. Hum. Genet..

[23] Mignot C., Apartis E., Durr A., Marques Lourenco C., Charles P., Devos D., Moreau C., de Lonlay P., Drouot N., Burglen L. (2013). Phenotypic variability in ARCA2 and identification of a core ataxic phenotype with slow progression. Orphanet J. Rare Dis..

[24] Gerards M., van den Bosch B., Calis C., Schoonderwoerd K., van Engelen K., Tijssen M., de Coo R., van der Kooi A., Smeets H. (2010). Nonsense mutations in *CABC1/ADCK3* cause progressive cerebellar ataxia and atrophy. Mitochondrion.

[25] Coutelier M., Coarelli G., Monin M., Konop J., Davoine C., Tesson C., Valter R., Anheim M., Behin A., Castelnovo G. (2017). A panel study on patients with dominant cerebellar ataxia highlights the frequency of channelopathies. Brain.

[26] Winkelmann J., Lin L., Schormair B., Kornum B.R., Faraco J., Plazzi G., Melberg A., Cornelio F., Urban A.E., Pizza F. (2012). Mutations in *DNMT1* cause autosomal dominant cerebellar ataxia, deafness and narcolepsy. Hum. Mol. Genet..

[27] Wan J., Yourshaw M., Mamsa H., Rudnik-schöneborn S., Menezes M.P., Hong J.E., Leong D.W., Senderek J., Salman M.S., Chitayat D. (2012). Mutations in the RNA exosome component gene *EXOSC3* cause pontocerebellar hypoplasia and spinal motor neuron degeneration. Nat. Genet..

[28] Galatolo D., Kuo M.E., Mullen P., Meyer-Schuman R., Doccini S., Battini R., Lieto M., Tessa A., Filla A., Francklyn C. (2020). Bi-allelic mutations in *HARS1* severely impair histidyl-tRNA synthetase expression and enzymatic activity causing a novel multisystem ataxic syndrome. Hum. Mutat..

[29] Möller G., Van Grunsven E.G., Wanders R.J.A., Adamski J. (2001). Molecular basis of D-bifunctional protein deficiency. Mol. Cell. Endocrinol..

[30] Barresi S., Niceta M., Alfieri P., Brankovich V., Piccini G., Bruselles A., Barone M., Cusmai R., Tartaglia M., Bertini E. (2017). Mutations in the IRBIT domain of *ITPR1* are a frequent cause of autosomal dominant nonprogressive congenital ataxia. Clin. Genet..

[31] Van Dijk E.L., Jaszczyszyn Y., Naquin D., Thermes C. (2018). The third revolution in sequencing technology. Trends Genet..

[32] Pena S., Coimbra R. (2015). Ataxia and myoclonic epilepsy due to a heterozygous new mutation in *KCNA2*: Proposal for a new channelopathy. Clin. Genet..

[33] Helbig K.L., Hedrich U.B.S., Shinde D.N., Krey I., Teichmann A., Hentschel J., Schubert J., Chamberlin A.C., Huether R., Lu H. (2016). A recurrent mutation in *KCNA2* as a novel cause of hereditary spastic paraplegia and ataxia. Ann. Neurol..

[34] Figueroa K.P., Minassian N.A., Stevanin G., Waters M., Garibyan V., Forlani S., Strzelczyk A., Bürk K., Brice A., Dürr A. (2010). *KCNC3*: Phenotype, mutations, channel biophysics—A study of 260 familial ataxia patients. Hum. Mutat..

[35] Hasdemir C., Juang J., Kose S., Kocabas U., Orman M., Payzin S., Sahin H., Celen C., Ozcan E., Chen C.Y.J.C. (2018). Co-existence of Atrioventricular Accessory Pathways and Drug-Induced Type 1 Brugada Pattern. Physiol. Behav..

[36] Lee Y., Durr A., Majczenko K., Huang Y., Liu Y., Lien C., Tsai P., Ichikawa Y., Goto J., Monin M.-L. (2012). Mutations in *KCND3* cause spinocerebellar ataxia type 22. Ann. Neurol..

[37] Nemani T., Steel D., Kaliakatsos M., DeVile C., Ververi A., Scott R., Getov S., Sudhakar S., Male A., Mankad K. (2020). *KIF1A*-related disorders in children: A wide spectrum of central and peripheral nervous system involvement. J. Peripher. Nerv. Syst..

[38] Micalizzi A., Poretti A., Romani M., Ginevrino M., Mazza T., Aiello C., Zanni G., Baumgartner B., Borgatti R., Brockmann K. (2016). Clinical, neuroradiological and molecular characterization of cerebellar dysplasia with cysts (Poretti-Boltshauser syndrome). Eur. J. Hum. Genet..

[39] Di Meglio C., Bonello-Palot N., Boulay C., Milh M., Ovaert C., Levy N., Chabrol B. (2016). Clinical and allelic heterogeneity in a pediatric cohort of 11 patients carrying *MFN2* mutation. Brain Dev..

[40] Lerner-Ellis J.P., Tirone J.C., Pawelek P.D., Doré C., Atkinson J.L., Watkins D., Morel C.F., Fujiwara T.M., Moras E., Hosack A.R. (2006). Identification of the gene responsible for methylmalonic aciduria and homocystinuria, cblC type. Nat. Genet..

[41] Nair P., Sabbagh S., Mansour H., Fawaz A., Hmaimess G., Noun P., Dagher R., Megarbane H., Hana S., Alame S. (2018). Contribution of next generation sequencing in pediatric practice in Lebanon. A Study on 213 cases. Mol. Genet. Genomic Med..

[42] Delettre C., Lenaers G., Griffoin J.M., Gigarel N., Lorenzo C., Belenguer P., Pelloquin L., Grosgeorge J., Turc-Carel C., Perret E. (2000). Nuclear gene *OPA1*, encoding a mitochondrial dynamin-related protein, is mutated in dominant optic atrophy. Nat. Genet..

[43] Wu Y., Jiang Y., Gao Z., Wang J., Yuan Y., Xiong H., Chang X., Bao X., Zhang Y., Xiao J. (2009). Clinical study and *PLA2G6* mutation screening analysis in Chinese patients with infantile neuroaxonal dystrophy. Eur. J. Neurol..

[44] Matthijs G., Schollen E., Pardon E., Veiga-Da-Cunha M., Jaeken J., Cassiman J., Van Schaftingen E. (1997). Mutations in *PMM2*, a phosphomannomutase gene on chromosome 16p13, in carbohydrate-deficient glycoprotein type I syndrome (Jaeken syndrome). Nat. Genet..

[45] Topaloglu A.K., Lomniczi A., Kretzschmar D., Dissen G.A., Kotan L.D., McArdle C.A., Koc A.F., Hamel B.C., Guclu M., Papatya E.D. (2014). Loss-of-function mutations in *PNPLA6* encoding neuropathy target esterase underlie pubertal failure and neurological deficits in Gordon Holmes syndrome. J. Clin. Endocrinol. Metab..

[46] Hufnagel R.B., Arno G., Hein N.D., Hersheson J., Prasad M., Anderson Y., Krueger L.A., Gregory L.C., Stoetzel C., Jaworek T.J. (2015). Neuropathy target esterase impairments cause Oliver-McFarlane and Laurence-Moon syndromes. J. Med. Genet..

[47] Synofzik M., Gonzalez M.A., Lourenco C.M., Coutelier M., Haack T.B., Rebelo A., Hannequin D., Strom T.M., Prokisch H., Lima-Martinez M.M. (2014). *PNPLA6* mutations cause Boucher-Neuhauser and Gordon Holmes syndromes as part of a broad neurodegenerative spectrum. Brain.

[48] Teive H.A.G., Camargo C.H.F., Sato M.T., Shiokawa N., Boguszewski C.L., Raskin S., Buck C., Seminara S.B., Munhoz R.P. (2018). Different cerebellar ataxia phenotypes associated with mutations of the *PNPLA6* gene in Brazilian patients with recessive ataxias. Cerebellum.

[49] Lamantea E., Tiranti V., Bordoni A., Toscano A., Bono F., Servidei S., Papadimitriou A., Spelbrink H., Silvestri L., Casari G. (2002). Mutations of mitochondrial DNA polymerase gammaA are a frequent cause of autosomal dominant or recessive progressive external ophthalmoplegia. Ann. Neurol..

[50] Van Goethem G., Schwartz M., Löfgren A., Dermaut B., Van Broeckhoven C., Vissing J. (2003). Novel *POLG* mutations in progressive external ophthalmoplegia mimicking mitochondrial neurogastrointestinal encephalomyopathy. Eur. J. Hum. Genet..

[51] Van Goethem G., Luoma P., Rantamäki M., Al Memar A., Kaakkola S., Hackman P., Krahe R., Löfgren A., Martin J.J., De Jonghe P. (2004). *POLG* mutations in neurodegenerative disorders with ataxia but no muscle involvement. Neurology.

[52] Minnerop M., Kurzwelly D., Wagner H., Soehn A.S., Reichbauer J., Tao F., Rattay T.W., Peitz M., Rehbach K., Giorgetti A. (2017). Hypomorphic mutations in *POLR3A* are a frequent cause of sporadic and recessive spastic ataxia. Brain.

[53] De Michele G., Galatolo D., Galosi S., Mignarri A., Silvestri G., Casali C., Leuzzi V., Ricca I., Barghigiani M., Tessa A. (2021). Episodic ataxia and severe infantile phenotype in spinocerebellar ataxia type 14: Expansion of the phenotype and novel mutations. J. Neurol..

[54] Vlak M.H.M., Sinke R.J., Rabelink G.M., Kremer B.P.H., van de Warrenburg B.P.C. (2006). Novel *PRKCG*/SCA14 mutation in a Dutch spinocerebellar ataxia family: Expanding the phenotype. Mov. Disord..

[55] Riso V., Rossi S., Perna A., Nicoletti T., Bosco L., Zanni G., Silvestri G. (2020). NGS-based detection of a novel mutation in *PRKCG* (SCA14) in sporadic adult-onset ataxia plus dystonic tremor. Neurol. Sci..

[56] Lieto M., Galatolo D., Roca A., Cocozza S., Pontillo G., Fico T., Pane C., Saccà F., De Michele G., Santorelli F.M. (2019). Overt hypogonadism may not be a sentinel sign of RING finger protein 216: Two novel mutations associated with ataxia, chorea, and fertility. Mov. Disord. Clin. Pract..

[57] Leen W.G., Mewasingh L., Verbeek M.M., Kamsteeg E.J., van de Warrenburg B.P., Willemsen M.A. (2013). Movement disorders in GLUT1 deficiency syndrome respond to the modified Atkins diet. Mov. Disord..

[58] Martinuzzi A., Montanaro D., Vavla M., Paparella G., Bonanni P., Musumeci O., Brighina E., Hlavata H., Rossi G., Aghakhanyan G. (2016). Clinical and paraclinical indicators of motor system impairment in hereditary spastic paraplegia: A pilot study. PLoS ONE.

[59] Coutelier M., Hammer M.B., Stevanin G., Monin M.L., Davoine C.S., Mochel F., Labauge P., Ewenczyk C., Ding J., Gibbs J.R. (2018). Efficacy of exome-targeted capture sequencing to detect mutations in known cerebellar ataxia genes. JAMA Neurol..

[60] Jacob F., Ho E.S., Martinez-ojeda M., Darras B.T., Khwaja O.S. (2013). Case of infantile onset spinocerebellar ataxia type 5. J. Child Neurol..

[61] De Michele G., Lieto M., Galatolo D., Salvatore E., Cocozza S., Barghigiani M., Tessa A., Baldacci J., Pappatà S., Filla A. (2019). Spinocerebellar ataxia 48 presenting with ataxia associated with cognitive, psychiatric, and extrapyramidal features: A report of two Italian families. Park. Relat. Disord..

[62] Depondt C., Donatello S., Simonis N., Rai M., van Heurck R., Abramowicz M., D’Hooghe M., Pandolfo M. (2014). Autosomal recessive cerebellar ataxia of adult onset due to *STUB1* mutations. Neurology.

[63] Lieto M., Riso V., Galatolo D., De Michele G., Rossi S., Barghigiani M., Cocozza S., Pontillo G., Trovato R., Saccà F. (2020). The complex phenotype of spinocerebellar ataxia type 48 in eight unrelated Italian families. Eur. J. Neurol..

[64] Genis D., Ortega-Cubero S., Nicolas H.S., Corral J., Gardenyes J., De Jorge L., Lopez E., Campos B., Lorenzo E., Tonda R. (2018). Heterozygous *STUB1* mutation causes familial ataxia with cognitive affective syndrome (SCA48). Neurology.

[65] Stamberger H., Nikanorova M., Willemsen M.H., Accorsi P., Angriman M., Baier H., Benkel-Herrenbrueck I., Benoit V., Budetta M., Caliebe A. (2016). *STXBP1* encephalopathy: A neurodevelopmental disorder including epilepsy. Neurology.

[66] Delplanque J., Devos D., Huin V., Genet A., Sand O., Moreau C., Goizet C., Charles P., Anheim M., Monin M.L. (2014). *TMEM240* mutations cause spinocerebellar ataxia 21 with mental retardation and severe cognitive impairment. Brain.

[67] Riso V., Galatolo D., Barghigiani M., Galosi S., Tessa A., Ricca I., Rossi S., Caputi C., Cioffi E., Leuzzi V. (2021). A next generation sequencing-based analysis of a large cohort of ataxic patients refines the clinical spectrum associated with spinocerebellar ataxia 21. Eur. J. Neurol..

[68] Sleat D.E., Gin R.M., Sohar I., Wisniewski K., Sklower-Brooks S., Pullarkat R.K., Palmer D.N., Lerner T.J., Boustany R.M., Uldall P. (1999). Mutational analysis of the defective protease in classic late-infantile neuronal ceroid lipofuscinosis, a neurodegenerative lysosomal storage disorder. Am. J. Hum. Genet..

[69] Rigoli L., Aloi C., Salina A., Di Bella C., Salzano G., Caruso R., Mazzon E., Maghnie M., Patti G., D’Annunzio G. (2020). Wolfram syndrome 1 in the Italian population: Genotype–Phenotype correlations. Pediatr. Res..

[70] Ohba C., Osaka H., Iai M., Yamashita S., Suzuki Y., Aida N., Shimozawa N., Takamura A., Doi H., Tomita-Katsumoto A. (2013). Diagnostic utility of whole exome sequencing in patients showing cerebellar and/or vermis atrophy in childhood. Neurogenetics.

[71] Sawyer S.L., Schwartzentruber J., Beaulieu C.L., Dyment D., Smith A., Chardon J.W., Yoon G., Rouleau G.A., Suchowersky O., Siu V. (2014). Exome sequencing as a diagnostic tool for pediatric-onset ataxia. Hum. Mutat..

[72] Fogel B., Lee H., Deignan J., Strom S., Kantarci S., Wang X., Quintero-Rivera F., Vilain E., Grody W., Perlman S. (2014). Exome sequencing in the clinical diagnosis of sporadic or familial cerebellar ataxia. JAMA Neurol..

[73] Pyle A., Smertenko T., Bargiela D., Griffin H., Duff J., Appleton M., Douroudis K., Pfeffer G., Santibanez-Koref M., Eglon G. (2015). Exome sequencing in undiagnosed inherited and sporadic ataxias. Brain.

[74] Keogh M.J., Steele H., Douroudis K., Pyle A., Duff J., Hussain R., Smertenko T., Griffin H., Santibanez-Koref M., Horvath R. (2015). Frequency of rare recessive mutations in unexplained late onset cerebellar ataxia. J. Neurol..

[75] Mallaret M., Renaud M., Redin C., Drouot N., Muller J., Severac F., Mandel J.L., Hamza W., Benhassine T., Ali-Pacha L. (2016). Validation of a clinical practice-based algorithm for the diagnosis of autosomal recessive cerebellar ataxias based on NGS identified cases. J. Neurol..

[76] van de Warrenburg B.P., Schouten M.I., de Bot S.T., Vermeer S., Meijer R., Pennings M., Gilissen C., Willemsen A.A.P., Scheffer H., Kamsteeg E.-J. (2016). Clinical exome sequencing for cerebellar ataxia and spastic paraplegia uncovers novel gene-disease associations and unanticipated rare disorders. Eur. J. Hum. Genet..

[77] Marelli C., Guissart C., Hubsch C., Renaud M., Villemin J.P., Larrieu L., Charles P., Ayrignac X., Sacconi S., Collignon P. (2016). Mini-exome coupled to read-depth based copy number variation analysis in patients with inherited ataxias. Hum. Mutat..

[78] Kuperberg M., Lev D., Blumkin L., Zerem A., Ginsberg M., Linder I., Carmi N., Kivity S., Lerman-Sagie T., Leshinsky-Silver E. (2016). Utility of whole exome sequencing for genetic diagnosis of previously undiagnosed pediatric neurology patients. J. Child Neurol..

[79] Hadjivassiliou M., Martindale J., Shanmugarajah P., Grünewald R.A., Sarrigiannis P.G., Beauchamp N., Garrard K., Warburton R., Sanders D.S., Friend D. (2017). Causes of progressive cerebellar ataxia: Prospective evaluation of 1500 patients. J. Neurol. Neurosurg. Psychiatry.

[80] Iqbal Z., Rydning S.L., Wedding I.M., Koht J., Pihlstrøm L., Rengmark A.H., Henriksen S.P., Tallaksen C.M.E., Toft M. (2017). Targeted high throughput sequencing in hereditary ataxia and spastic paraplegia. PLoS ONE.

[81] Nibbeling E.A.R., Duarri A., Verschuuren-Bemelmans C.C., Fokkens M.R., Karjalainen J.M., Smeets C.J.L.M., De Boer-Bergsma J.J., Van Der Vries G., Dooijes D., Bampi G.B. (2017). Exome sequencing and network analysis identifies shared mechanisms underlying spinocerebellar ataxia. Brain.

[82] Montaut S., Tranchant C., Drouot N., Rudolf G., Guissart C., Tarabeux J., Stemmelen T., Velt A., Fourrage C., Nitschké P. (2018). Assessment of a targeted gene panel for identification of genes associated with movement disorders. JAMA Neurol..

[83] Dong H.L., Ma Y., Li Q.F., Du Y.C., Yang L., Chen S., Wu Z.Y. (2019). Genetic and clinical features of Chinese patients with mitochondrial ataxia identified by targeted next-generation sequencing. CNS Neurosci. Ther..

[84] Kang C., Liang C., Ahmad K.E., Gu Y., Siow S.F., Colebatch J.G., Whyte S., Ng K., Cremer P.D., Corbett A.J. (2019). High degree of genetic heterogeneity for hereditary cerebellar ataxias in Australia. Cerebellum.

[85] Shakya S., Kumari R., Suroliya V., Tyagi N., Joshi A., Garg A., Singh I., Kalikavil Puthanveedu D., Cherian A., Mukerji M. (2019). Whole exome and targeted gene sequencing to detect pathogenic recessive variants in early onset cerebellar ataxia. Clin. Genet..

[86] Sun M., Johnson A.K., Nelakuditi V., Guidugli L., Fischer D., Arndt K., Ma L., Sandford E., Shakkottai V., Boycott K. (2019). Targeted exome analysis identifies the genetic basis of disease in over 50% of patients with a wide range of ataxia-related phenotypes. Genet. Med..

[87] Arslan E.A., Öncel İ., Ceylan A.C., Topçu M., Topaloğlu H. (2020). Genetic and phenotypic features of patients with childhood ataxias diagnosed by next-generation sequencing gene panel. Brain Dev..

[88] Ngo K.J., Rexach J.E., Lee H., Petty L.E., Perlman S., Valera J.M., Deignan J.L., Mao Y., Aker M., Posey J.E. (2020). A diagnostic ceiling for exome sequencing in cerebellar ataxia and related neurological disorders. Hum. Mutat..

[89] Mutlu-Albayrak H., Kırat E., Gürbüz G. (2020). Childhood-onset autosomal recessive ataxias: A cross-sectional study from Turkey. Neurogenetics.

[90] Gauquelin L., Hartley T., Tarnopolsky M., Dyment D.A., Brais B., Geraghty M.T., Tétreault M., Ahmed S., Rojas S., Choquet K. (2020). Channelopathies are a frequent cause of genetic ataxias associated with cerebellar atrophy. Mov. Disord. Clin. Pract..

[91] Ignatius E., Isohanni P., Pohjanpelto M., Lahermo P., Ojanen S., Brilhante V., Palin E., Suomalainen A., Lönnqvist T., Carroll C.J. (2020). Genetic background of ataxia in children younger than 5 years in Finland. Neurol. Genet..

[92] Kim M., Kim A.R., Kim J.S., Park J., Youn J., Ahn J.H., Mun J.K., Lee C., Kim N.S., Kim N.K.D. (2020). Clarification of undiagnosed ataxia using whole-exome sequencing with clinical implications. Park. Relat. Disord..

[93] Bogdanova-Mihaylova P., Hebert J., Moran S., Murphy M., Ward D., Walsh R.A., Murphy S.M. (2021). Inherited cerebellar ataxias: 5-year experience of the Irish National Ataxia Clinic. Cerebellum.

[94] Synofzik M., Németh A. (2018). Recessive ataxias. Handb. Clin. Neurol..

[95] Roux T., Barbier M., Papin M., Davoine C.S., Sayah S., Coarelli G., Charles P., Marelli C., Parodi L., Tranchant C. (2020). Clinical, neuropathological, and genetic characterization of *STUB1* variants in cerebellar ataxias: A frequent cause of predominant cognitive impairment. Genet. Med..

[96] Ravel J.M., Benkirane M., Calmels N., Marelli C., Ory-Magne F., Ewenczyk C., Halleb Y., Tison F., Lecocq C., Pische G. (2021). Expanding the clinical spectrum of STIP1 homology and U-box containing protein 1-associated ataxia. J. Neurol..

[97] Coarelli G., Schule R., van de Warrenburg B., De Jonghe P., Ewenczyk C., Martinuzzi A., Synofzik M., Hamer E., Baets J., Anheim M. (2019). Loss of paraplegin drives spasticity rather than ataxia in a cohort of 241 patients with SPG7. Neurology.

[98] Mancini C., Giorgio E., Rubegni A., Pradotto L., Bagnoli S., Rubino E., Prontera P., Cavalieri S., Di Gregorio E., Ferrero M. (2019). Prevalence and phenotype of the c.1529C>T *SPG7* variant in adult-onset cerebellar ataxia in Italy. Eur. J. Neurol..

[99] Rainier S., Bui M., Mark E., Thomas D., Tokarz D., Ming L., Delaney C., Richardson R.J., Albers J.W., Matsunami N. (2008). Neuropathy target esterase gene mutations cause motor neuron disease. Am. J. Hum. Genet..

[100] Traschütz A., van Gaalen J., Oosterloo M., Vreeburg M., Kamsteeg E.J., Deininger N., Rieß O., Reimold M., Haack T., Schöls L. (2019). The movement disorder spectrum of SCA21 (ATX-*TMEM240*): 3 novel families and systematic review of the literature. Park. Relat. Disord..

[101] Synofzik M., Puccio H.M., Mochel F., Schols L. (2019). Autosomal recessive cerebellar ataxias: Paving the way toward targeted molecular therapies. Neuron.

[102] van Dijk T., Barth P., Reneman L., Appelhof B., Baas F., Poll-The B.T. (2017). A de novo missense mutation in the inositol 1,4,5-triphosphate receptor type 1 gene causing severe pontine and cerebellar hypoplasia: Expanding the phenotype of ITPR1-related spinocerebellar ataxias. Am. J. Med. Genet. Part A.

[103] Gonzalez M., Falk M.J., Gai X., Postrel R., Schule R., Zuchner S. (2015). Innovative genomic collaboration using the GENESIS (GEM.app) platform. Hum. Mutat..

[104] Stitziel N.O., Kiezun A., Sunyaev S. (2011). Computational and statistical approaches to analyzing variants identified by exome sequencing. Genome Biol..

[105] Riso V., Rossi S., Nicoletti T.F., Tessa A., Travaglini L., Zanni G., Aiello C., Perna A., Barghigiani M., Pomponi M.G. (2021). Application of a clinical workflow may lead to increased diagnostic precision in hereditary spastic paraplegias and cerebellar ataxias: A single center experience. Brain Sci..

[106] Baroni M.G., Oelbaum R.S., Pozzilli P., Stocks J., Li S.-R., Fiore V., Galton D.J. (1992). Polymorphisms at the GLUT1 (HepG2) and GLUT4 (muscle/adipocyte) glucose transporter genes and non-insulin-dependent diabetes mellitus (NIDDM). Hum. Genet..

[107] Cocozza S., Pontillo G., De Michele G., Perillo T., Guerriero E., Ugga L., Salvatore E., Galatolo D., Riso V., Saccà F. (2020). The “crab sign”: An imaging feature of spinocerebellar ataxia type 48. Neuroradiology.

[108] Marques Matos C., Alonso I., Leão M. (2019). Diagnostic yield of next-generation sequencing applied to neurological disorders. J. Clin. Neurosci..

[109] Verdura E., Schlüter A., Fernández-Eulate G., Ramos-Martín R., Zulaica M., Planas-Serra L., Ruiz M., Fourcade S., Casasnovas C., López de Munain A. (2020). A deep intronic splice variant advises reexamination of presumably dominant SPG7 cases. Ann. Clin. Transl. Neurol..

[110] Magri S., Fracasso V., Plumari M., Alfei E., Ghezzi D., Gellera C., Rusmini P., Poletti A., Di Bella D., Elia A. (2018). Concurrent *AFG3L2* and *SPG7* mutations associated with syndromic parkinsonism and optic atrophy with aberrant OPA1 processing and mitochondrial network fragmentation. Hum. Mutat..

[111] Bis-Brewer D.M., Gan-Or Z., Sleiman P., Rodriguez A., Bacha A., Kosikowski A., Wood B., McCray B., Blume B., Siskind C. (2020). Assessing non-Mendelian inheritance in inherited axonopathies. Genet. Med..

[112] Warner J.P., Barron L.H., Goudie D., Kelly K., Dow D., Fitzpatrick D.R., Brock D.J. (1996). A general method for the detection of large CAG repeat expansions by fluorescent PCR. J. Med. Genet. Genet..

[113] Cagnoli C., Stevanin G., Michielotto C., Promis G.G., Brussino A., Pappi P., Durr A., Dragone E., Viemont M., Gellera C. (2006). Large pathogenic expansions in the SCA2 and SCA7 genes can be detected by fluorescent repeat-primed polymerase chain reaction assay. J. Mol. Diagn..

[114] Campuzano V., Montermini L., Molto M.D., Pianese L., Cossee M., Cavalcanti F., Monros E., Duclos F., Monticelli A., Zara F. (1996). Friedreich’s ataxia: Autosomal recessive disease caused by an intronic GAA triplet repeat expansion. Science.

[115] Richards S., Aziz N., Bale S., Bick D., Das S., Gastier-Foster J., Grody W.W., Hegde M., Lyon E., Spector E. (2015). Standards and guidelines for the interpretation of sequence variants: A joint consensus recommendation of the American College of Medical Genetics and Genomics and the Association for Molecular Pathology. Genet. Med..

[116] Rodrigues C.H.M., Pires D.E.V., Ascher D.B. (2018). DynaMut: Predicting the impact of mutations on protein conformation, flexibility and stability. Nucleic Acids Res..

[117] Frappier V., Najmanovich R.J. (2014). A Coarse-Grained Elastic Network Atom Contact Model and Its Use in the Simulation of Protein Dynamics and the Prediction of the Effect of Mutations. PLoS Comput. Biol..

[118] Pires D.E.V., Ascher D.B., Blundell T.L. (2014). MCSM: Predicting the effects of mutations in proteins using graph-based signatures. Bioinformatics.

[119] Pandurangan A.P., Ochoa-Montaño B., Ascher D.B., Blundell T.L. (2017). SDM: A server for predicting effects of mutations on protein stability. Nucleic Acids Res..

[120] Pires D.E.V., Ascher D.B., Blundell T.L. (2014). DUET: A server for predicting effects of mutations on protein stability using an integrated computational approach. Nucleic Acids Res..

