# Supplementary material for: NGS in Hereditary Ataxia: When Rare Becomes Frequent
Source: Int J Mol Sci. 2021 Aug 6;22(16):8490. doi: 10.3390/ijms22168490 (PMC8395181; doi:10.3390/ijms22168490)
Supplement: Supplementary file 1 [file ijms-22-08490-s001.zip › Table S2, S3, S9-FiguresS1-S2.pdf]

Table S2. Protein domain localization analysis of novel missense variants of pathogenic significance.

Gene (Ref_Seq)	cDNA Variant	Protein Variant	UniProt Code	Protein Domain Localization
ABCD1 (NM_000033.4)	c.2087A>T	p.Lys696Met	P33897	ATP-binding domain [1]
AFG3L2 (NM_006796.3)	c.1712T>G	p.Val571Gly	Q9Y4W6	Proteolytic domain [2]
ATM (NM_000051.3)	c.2929T>C	p.Cys977Arg	Q13315	Unspecific region [3]
CACNA1A (NM_001127222.2)	c.4897G>A	p.Asp1633Asn	O00555	Transmembrane helix S3 of repeat domain IV [4]
CACNA1A (NM_001127222.2)	c.4927G>A	p.Asp1643Asn	O00555	Transmembrane helix S3 of repeat domain IV [4]
CACNA1A (NM_001127222.2)	c.4466T>C	p.Ile1489Thr	O00555	Transmembrane helix S6 of repeat domain III [4]
CACNA1G (NM_018896.5)	c.481A>T	p.Ile161Phe	O43497	Transmembrane helix S3 of repeat domain I [5]
CACNA1G (NM_018896.5)	c.3835G>A	p.Asp1279Asn	O43497	Transmembrane helix S1 of repeat domain III [5]
COQ8A (NM_020247.5)	c.1376T>C	p.Leu459Pro	Q8NI60	C lobe insert, unclear function [6]
ERCC4 (NM_005236.3)	c.2248C>T	p.Arg750Cys	Q92889	Nuclease domain [7]
GJC2 (NM_020435.4)	c.254T>C	p.Val85Ala	Q5T442	Cytoplasmic unspecific domain [8]
ITPR1 (NM_001099952.3)	c.2816G>A	p.Gly939Glu	Q14643	Coupling/regulatory region [30]
KCND3 (NM_004980.4)	c.611C>T	p.Thr204Met	Q9UK17	Transmembrane helix S2 [36]
MME (NM_007288.3)	c.2154G>T	p.Arg718Ser	P08473	M13 peptidase [9]
OPA1 (NM_130837.2)	c.885C>G	p.Asn295Lys	O60313	GTPase domain [10]
PLA2G6 (NM_003560.4)	c.1703T>C	p.Phe568Ser	O60733	Patatin-like phospholipase domain [11]
PNPLA6 (NM_001166111.2)	c.1880C>T	p.Ala627Val	Q8IY17	Cyclic nucleotide binding sites [12]
PNPLA6 (NM_001166111.2)	c.2264A>C	p.Gln755Pro	Q8IY17	Cyclic nucleotide binding sites [12]
PNPLA6 (NM_001166111.2)	c.3388C>T	p.His1130Tyr	Q8IY17	Phospholipid esterase domain [12]
PNPLA6 (NM_001166111.2)	c.3023A>G	p.Asp1008Gly	Q8IY17	Phospholipid esterase domain [12]
POLR3A (NM_007055.4)	c.4073G>A	p.Gly1358Glu	O14802	Clamp core [13]
PRNP (NM_001080123.3)	c.305C>T	p.Pro102Leu	P04156	Proteinase K resistant core [14]
RARS2 (NM_020320.5)	c.517G>A	p.Asp173Asn	Q5T160	Catalytic domain [15]
RARS2 (NM_020320.5)	c.1037C>T	p.Thr346Ile	Q5T160	Catalytic domain [15]
RNF170 (NM_030954.4)	c.566T>G	p.Phe189Cys	Q96K19	Cytoplasmic unspecific domain [16]
SETX (NM_015046.7)	c.5591A>C	p.Gln1864Pro	Q7Z333	Unspecific region [17]
SLC2A1 (NM_006516.3)	c.985G>A	p.Glu329Lys	P11166	Cytoplasmatic region between TM domains [18]
SPG7 (NM_003119.4)	c.1940C>A	p.Ala647Glu	Q9UQ90	M41 peptidase [19]
SPTAN1 (NM_001363759.2)	c.4870C>T	p.Arg1624Cys	Q13813	Spectrin repeats [20]
SPTBN2 (NM_006946.3)	c.5066G>A	p.Arg1689His	O15020	Spectrin repeats [21]
SPTBN2 (NM_006946.3)	c.1843C>T	p.Arg615Trp	O15020	Spectrin repeats [21]
STXBP1 (NM_003165.5)	c.434A>G	p.Tyr145Cys	P61764	Domain 2, unclear function [22]
STXBP1 (NM_003165.5)	c.298C>T	p.Arg100Trp	P61764	Domain 1, unclear function [22]
TMEM240 (NM_001114748.1)	c.419T>A	p.Leu140Gln	Q5SV17	Intracellular unspecific domain [66]
TTBK2 (NM_173500.4)	c.239T>A	p.Phe80Tyr	Q6IQ55	Kinase domain [23]
WFS1 (NM_006005.3)	c.1291G>C	p.Glu431Gln	O76024	Transmembrane domain [24]

References

1. Emamalizadeh, B.; Daneshmandpour, Y.; Tafakhori, A.; Ranji-Burachaloo, S.; Shafiee, S.; Ghods, E.; Darvish, H. Novel *ABCD1* gene mutations in Iranian pedigrees with X-linked adrenoleukodystrophy. *J. Pediatr. Endocrinol. Metab.* **2019**, *32*, 1207–1215, doi:10.1515/jpem-2019-0244.
2. Caporali, L.; Magri, S.; Legati, A.; Del Dotto, V.; Tagliavini, F.; Balistreri, F.; Nasca, A.; La Morgia, C.; Carbonelli, M.; Valentino, M.L.; et al. ATPase Domain AFG3L2 Mutations Alter OPA1 Processing and Cause Optic Neuropathy. *Ann. Neurol.* **2020**, *88*, 18–32, doi:10.1002/ana.25723.
3. Jette, N.; Lees-Miller, S.P. The DNA-dependent protein kinase: a multifunctional protein kinase with roles in DNA double strand break repair and mitosis. *Prog. Biophys. Mol. Biol.* **2015**, *117*, 194–205, doi:10.1016/j.pbiomolbio.2014.12.003.
4. Stendel, C.; D'Adamo, M.C.; Wiessner, M.; Dusl, M.; Cenciarini, M.; Belia, S.; Nematian-Ardestani, E.; Bauer, P.; Senderek, J.; Klopstock, T.; et al. Association of a novel splice site mutation in P/Q-type calcium channels with childhood epilepsy and late-onset slowly progressive non-episodic cerebellar ataxia. *Int. J. Mol. Sci.* **2020**, *21*, 3810, doi:10.3390/ijms21113810.
5. Weiss, N.; Zamponi, G.W. Genetic T-type calcium channelopathies. *J. Med. Genet.* **2020**, *57*, 1–10, doi:10.1136/jmedgenet-2019-106163.
6. Traschütz, A.; Schirinzi, T.; Laugwitz, L.; Murray, N.H.; Bingman, C.A.; Reich, S.; Kern, J.; Heinzmann, A.; Vasco, G.; Bertini, E.; et al. Clinico-Genetic, Imaging and Molecular Delineation of *COQ8A*-Ataxia: A Multicenter Study of 59 Patients. *Ann. Neurol.* **2020**, *88*, 251–263, doi:10.1002/ana.25751.
7. Mori, T.; Yousefzadeh, M.J.; Faridounnia, M.; Chong, J.X.; Hisama, F.M.; Hudgins, L.; Mercado, G.; Wade, E.A.; Barghouthy, A.S.; Lee, L.; et al. *ERCC4* variants identified in a cohort of patients with segmental progeroid syndromes. *Hum. Mutat.* **2018**, *39*, 255–265, doi:10.1002/humu.23367.
8. Kuipers, D.J.S.; Tufekcioglu, Z.; Bilgiç, B.; Olgiati, S.; Dremmen, M.H.G.; van IJcken, W.F.J.; Breedveld, G.J.; Mancini, G.M.S.; Hanagasi, H.A.; Emre, M.; et al. Late-onset phenotype associated with a homozygous *GJC2* missense mutation in a Turkish family. *Park. Relat. Disord.* **2019**, *66*, 228–231, doi:10.1016/j.parkreldis.2019.07.033.
9. Depondt, C.; Donatello, S.; Rai, M.; Wang, F.C.; Manto, M.; Simonis, N.; Pandolfo, M. *MME* mutation in dominant spinocerebellar ataxia with neuropathy (SCA43). *Neurol. Genet.* **2016**, *2*, e94, doi:10.1212/NXG.0000000000000094.
10. Yan, L.; Qi, Y.; Ricketson, D.; Li, L.; Subramanian, K.; Zhao, J.; Yu, C.; Wu, L.; Sarsam, R.; Wong, M.; et al. Structural analysis of a trimeric assembly of the mitochondrial dynamin-like GTPase Mgm1. *Proc. Natl. Acad. Sci. U. S. A.* **2020**, *117*, 4061–4070, doi:10.1073/pnas.1919116117.
11. Tsuboi, M.; Watanabe, M.; Nibe, K.; Yoshimi, N.; Kato, A.; Sakaguchi, M.; Yamato, O.; Tanaka, M.; Kuwamura, M.; Kushida, K.; et al. Identification of the *PLA2G6* c.1579G>A missense mutation in papillon dog neuroaxonal dystrophy using whole exome sequencing analysis. *PLoS One* **2017**, *12*, e0169002, doi:10.1371/journal.pone.0169002.
12. Sunderhaus, E.R.; Law, A.D.; Kretzschmar, D. Disease-Associated *PNPLA6* Mutations Maintain Partial Functions When Analyzed in Drosophila. *Front. Neurosci.* **2019**, *13*, 1207, doi:10.3389/fnins.2019.01207.
13. Arimbasseri, A.G.; Maraia, R.J. RNA Polymerase III Advances: Structural and tRNA Functional Views. *Trends Biochem. Sci.* **2016**, *41*, 546–559, doi:10.1016/j.tibs.2016.03.003.
14. Kim, Y.; Won, S.; Jeong, B. Identification of Prion Disease-Related Somatic Mutations in the Prion Protein Gene (*PRNP*) in Cancer Patients. *Cells* **2020**, *9*, 1480, doi:10.3390/cells9061480.
15. Nevanlinna, V.; Konovalova, S.; Ceulemans, B.; Muona, M.; Laari, A.; Hilander, T.; Gorski, K.; Valanne, L.; Anttonen, A.K.; Tyynismaa, H.; et al. A patient with pontocerebellar hypoplasia type 6: Novel *RARS2* mutations, comparison to previously published patients and clinical distinction from PEHO syndrome. *Eur. J. Med. Genet.* **2020**, *63*, 103766, doi:10.1016/j.ejmg.2019.103766.
16. Lu, J.P.; Wang, Y.; Sliter, D.A.; Pearce, M.M.P.; Wojcikiewicz, R.J.H. *RNF170* protein, an endoplasmic reticulum membrane ubiquitin ligase, mediates inositol 1,4,5-trisphosphate receptor ubiquitination and degradation. *J. Biol. Chem.* **2011**, *286*, 24426–24433, doi:10.1074/jbc.M111.251983.
17. Tripolszki, K.; Török, D.; Goudenège, D.; Farkas, K.; Sulák, A.; Török, N.; Engelhardt, J.I.; Klivényi, P.; Procaccio, V.; Nagy, N.; et al. High-throughput sequencing revealed a novel *SETX* mutation in a Hungarian patient with amyotrophic lateral sclerosis. *Brain Behav.* **2017**, *7*, e00669, doi:10.1002/brb3.669.
18. Suls, A.; Dedeken, P.; Goffin, K.; Van Esch, H.; Dupont, P.; Cassiman, D.; Kempfle, J.; Wuttke, T. V.; Weber, Y.; Lerche, H.; et al. Paroxysmal exercise-induced dyskinesia and epilepsy is due to mutations in *SLC2A1*, encoding the glucose transporter GLUT1. *Brain* **2008**, *131*, 1831–1844, doi:10.1093/brain/awn113.
19. Hewamadduma, C.A.; Hoggard, N.; O'Malley, R.; Robinson, M.; Beauchamp, N.; Segamogaite, R.; Martindale, J.; Rodgers, T.; Rao, G.; Sarrigiannis, P.; et al. Novel genotype-phenotype and MRI correlations in a large cohort of patients with *SPG7* mutations. *Neurol. Genet.* **2018**, *4*, e279, doi:10.1212/NXG.0000000000000279.
20. Beijer, D.; Deconinck, T.; de Bleecker, J.L.; Dotti, M.T.; Malandrini, A.; Andoni Urtizberea, J.; Zulaica, M.; de Munain, A.L.; Asselbergh, B.; de Jonghe, P.; et al. Nonsense mutations in alpha-II spectrin in three families with juvenile onset hereditary motor neuropathy. *Brain* **2019**, *142*, 2605–2616, doi:10.1093/brain/awz216.
21. Nicita, F.; Nardella, M.; Bellacchio, E.; Alfieri, P.; Terrone, G.; Piccini, G.; Graziola, F.; Pignata, C.; Capuano, A.; Bertini, E.; et al. Heterozygous missense variants of *SPTBN2* are a frequent cause of congenital cerebellar ataxia. *Clin. Genet.* **2019**, *96*, 169–175, doi:10.1111/cge.13562.

22. Uddin, M.; Woodbury-Smith, M.; Chan, A.J.S.; Albanna, A.; Minassian, B.; Boelman, C.; Scherer, S.W. Genomic context analysis of de novo *STXBP1* mutations identifies evidence of splice site DNA-motif associated hotspots. *G3 Genes, Genomes, Genet.* **2018**, *8*, 1115–1118, doi:10.1534/g3.118.200080.
23. Taylor, L.M.; McMillan, P.J.; Kraemer, B.C.; Liachko, N.F. Tau tubulin kinases in proteinopathy. *FEBS J.* **2019**, *286*, 2434–2446, doi:10.1111/febs.14866.
24. Qian, X.; Qin, L.; Xing, G.; Cao, X. Phenotype prediction of pathogenic nonsynonymous single nucleotide polymorphisms in *WFS1*. Sci. Rep. **2015**, 5, 14731, doi:10.1038/srep14731.

Table S3. Computational analysis of protein stability changes.

Gene (Ref_Seq)	Protein Variant	PDB Code	DynaMut ΔΔG Prediction	ENCoM ΔΔG Prediction	mCSM ΔΔG Prediction	SDM ΔΔG Prediction	DUET ΔΔG Prediction
STUB1 (NM_005861.4)	p.Gly33Ser	6NSV	−1.446 kcal/mol (Destabilizing)	0.587 kcal/mol (Stabilizing)	−2.003 kcal/mol (Destabilizing)	−2.270 kcal/mol (Destabilizing)	−2.142 kcal/mol (Destabilizing)
STUB1 (NM_005861.4)	p.Ala67Thr	6NSV	−1.891 kcal/mol (Destabilizing)	0.212 kcal/mol (Destabilizing)	−1.882 kcal/mol (Destabilizing)	−2.810 kcal/mol (Destabilizing)	−2.110 kcal/mol (Destabilizing)
STUB1 (NM_005861.4)	p.Lys145Gln	6NSV	−0.968 kcal/mol (Destabilizing)	−0.367 kcal/mol (Destabilizing)	−0.941 kcal/mol (Destabilizing)	−0.310 kcal/mol (Destabilizing)	−0.706 kcal/mol (Destabilizing)
STUB1 (NM_005861.4)	p.Pro57Leu	6NSV	0.797 kcal/mol (Stabilizing)	0.234 kcal/mol (Destabilizing)	−0.218 kcal/mol (Destabilizing)	0.840 kcal/mol (Stabilizing)	0.195 kcal/mol (Stabilizing)
AFG3L2 (NM_006796.3)	p.Val571Gly	6NYY	1.850 kcal/mol (Stabilizing)	4.195 kcal/mol (Stabilizing)	0.000 kcal/mol (Stabilizing)	0.000 kcal/mol (Stabilizing)	0.000 kcal/mol (Stabilizing)
ATM (NM_000051.3)	p.Cys977Arg	6K9K	1.492 kcal/mol (Stabilizing)	4.375 kcal/mol (Stabilizing)	0.457 kcal/mol (Stabilizing)	0.000 kcal/mol (Stabilizing)	0.000 kcal/mol (Stabilizing)
CACNA1G (NM_018896.5)	p.Ile161Phe	6KZO	0.888 kcal/mol (Stabilizing)	0.403 kcal/mol (Destabilizing)	−0.957 kcal/mol (Destabilizing)	−0.450 kcal/mol (Destabilizing)	−0.941 kcal/mol (Destabilizing)
COQ8A (NM_020247.5)	p.Leu459Pro	5I35	0.582 kcal/mol (Stabilizing)	0.616 kcal/mol (Stabilizing)	−0.777 kcal/mol (Destabilizing)	0.080 kcal/mol (Stabilizing)	−0.591 kcal/mol (Destabilizing)
ERCC4 (NM_005236.3)	p.Arg750Cys	6SXB	1.153 kcal/mol (Stabilizing)	4.139 kcal/mol (Stabilizing)	−0.277 kcal/mol (Destabilizing)	−0.330 kcal/mol (Destabilizing)	−0.328 kcal/mol (Destabilizing)
POLR3A (NM_007055.4)	p.Gly1358Glu	7AST	2.240 kcal/mol (Stabilizing)	4.958 kcal/mol (Stabilizing)	−0.306 kcal/mol (Destabilizing)	0.260 kcal/mol (Stabilizing)	0.022 kcal/mol (Stabilizing)
SLC2A1 (NM_006516.3)	p.Glu329Lys	6THA	−0.020 kcal/mol (Destabilizing)	0.058 kcal/mol (Destabilizing)	0.248 kcal/mol (Stabilizing)	−0.380 kcal/mol (Destabilizing)	0.586 kcal/mol (Stabilizing)

Table S9. Full list of genes (Ataxome 4.0, SureSelect, Agilent Technologies) available in the TRP employed in this study.

AARS2	*ABCB7*	*ABCD1*
ABHD12	*ACO2*	*ADGRG1*
AFG3L2	*AHDC1*	*AHI1*
ALDH5A1	*ALG3*	*ALG6*
AMACR	*AMPD2*	*ANO10*
APOB	*APTX*	*ARL13B*
ARSA	*ATCAY*	*ATG5*
ATL1	*ATM*	*ATP13A2*
ATP1A2	*ATP1A3*	*ATP2B3*
ATP7B	*ATP8A2*	*BEAN1*
BRAT1	*BRF1*	*C10ORF2*
C12ORF65	*C19ORF12*	*C5ORF42*
C9ORF72	*CA8*	*CACNA1A*
CACNA1G	*CACNB4*	*CAMTA1*
CASK	*CC2D2A*	*CCDC88C*
CD40LG	*CDK5*	*CEP104*
CEP290	*CEP41*	*CHMP1A*
CLCN2	*CLN5*	*CLN6*
CLN8	*CLP1*	*COA7*
COQ2	*COQ4*	*COQ8A*
COQ9	*COX20*	*CP*
CSPP1	*CSTB*	*CTBP1*
CTSD	*CWF19L1*	*CYP27A1*
CYP7B1	*DAB1*	*DARS*
DARS2	*DDHD2*	*DKC1*
DNAJC19	*DNAJC3*	*DNMT1*
EEF2	*EIF2B1*	*EIF2B2*
EIF2B3	*EIF2B4*	*EIF2B5*
ELOVL4	*ELOVL5*	*ERCC4*
ERCC8	*EXOSC3*	*EXOSC8*
FA2H	*FARS2*	*FASTKD2*
FAT2	*FGF14*	*FLVCR1*
FMR1	*FOLR1*	*FXN*
GALC	*GAN*	*GBA2*
GBE1	*GFAP*	*GJB1*
GJC2	*GLB1*	*GOSR2*
GRID2	*GRM1*	*HARS*
HARS2	*HEXA*	*HEXB*
HIBCH	*HSD17B4*	*INPP5E*
ITPR1	*KCNA1*	*KCNA2*
KCNC1	*KCNC3*	*KCND3*
KCNJ10	*KCNMA1*	*KCTD7*
KIF1A	*KIF1C*	*KIF7*
LAMA1	*LMNB2*	*LYST*
MARS2	*MED17*	*MFN2*
MFSD8	*MKS1*	*MMACHC*
MME	*MRE11A*	*MTPAP*
MTTP	*MVK*	*NAGLU*
NDUFS1	*NDUFS7*	*NEU1*
NOL3	*NOP56*	*NPC1*

NPC2	*NPHP1*	*OFD1*
OPA1	*OPA3*	*OPHN1*
PAX6	*PCLO*	*PCNA*
PDE6D	*PDHA1*	*PDSS1*
PDSS2	*PDYN*	*PEX10*
PEX16	*PEX2*	*PEX6*
PEX7	*PHYH*	*PIK3R5*
PLA2G6	*PLD3*	*PLP1*
PMM2	*PMPCA*	*PNKP*
PNPLA6	*POLG*	*POLR3A*
POLR3B	*PPT1*	*PRICKLE1*
PRKCG	*PRNP*	*PRPS1*
PRRT2	*PSAP*	*PSEN1*
PTF1A	*PTRH2*	*PUM1*
QARS	*RAB3GAP1*	*RARS*
RARS2	*RELN*	*RNF170*
RNF216	*RPGRIP1L*	*RUBCN*
SACS	*SAMD9L*	*SARS*
SCN1A	*SCN2A*	*SCN8A*
SCYL1	*SEPSECS*	*SETX*
SIL1	*SLC17A5*	*SLC1A3*
SLC25A46	*SLC2A1*	*SLC33A1*
SLC35A2	*SLC52A2*	*SLC6A19*
SLC9A1	*SLC9A6*	*SMPD1*
SNAP25	*SNX14*	*SPAST*
SPG11	*SPG7*	*SPTAN1*
SPTBN2	*SRD5A3*	*STS*
STUB1	*STXBP1*	*SURF1*
SYNE1	*SYNE2*	*SYT14*
TBC1D23	*TCTN1*	*TCTN2*
TCTN3	*TDP1*	*TDP2*
TGM6	*TINF2*	*TMEM138*
TMEM216	*TMEM231*	*TMEM237*
TMEM240	*TMEM67*	*TOE1*
TOP1	*TPP1*	*TRNT1*
TRPC3	*TSEN15*	*TSEN2*
TSEN34	*TSEN54*	*TTBK2*
TTC19	*TTC21B*	*TTPA*
TUBB3	*TUBB4A*	*UBA5*
UBR4	*UCHL1*	*VAMP1*
VARS2	*VLDLR*	*VPS53*
VRK1	*VWA3B*	*WDR73*
WDR81	*WFS1*	*WWOX*
XPA	*XRCC1*	*XRCC4*
ZFYVE26	*ZFYVE27*	*ZNF423*

.

Figure S1. Conservation analysis through species of novel variants of pathogenic significance identified in this study.

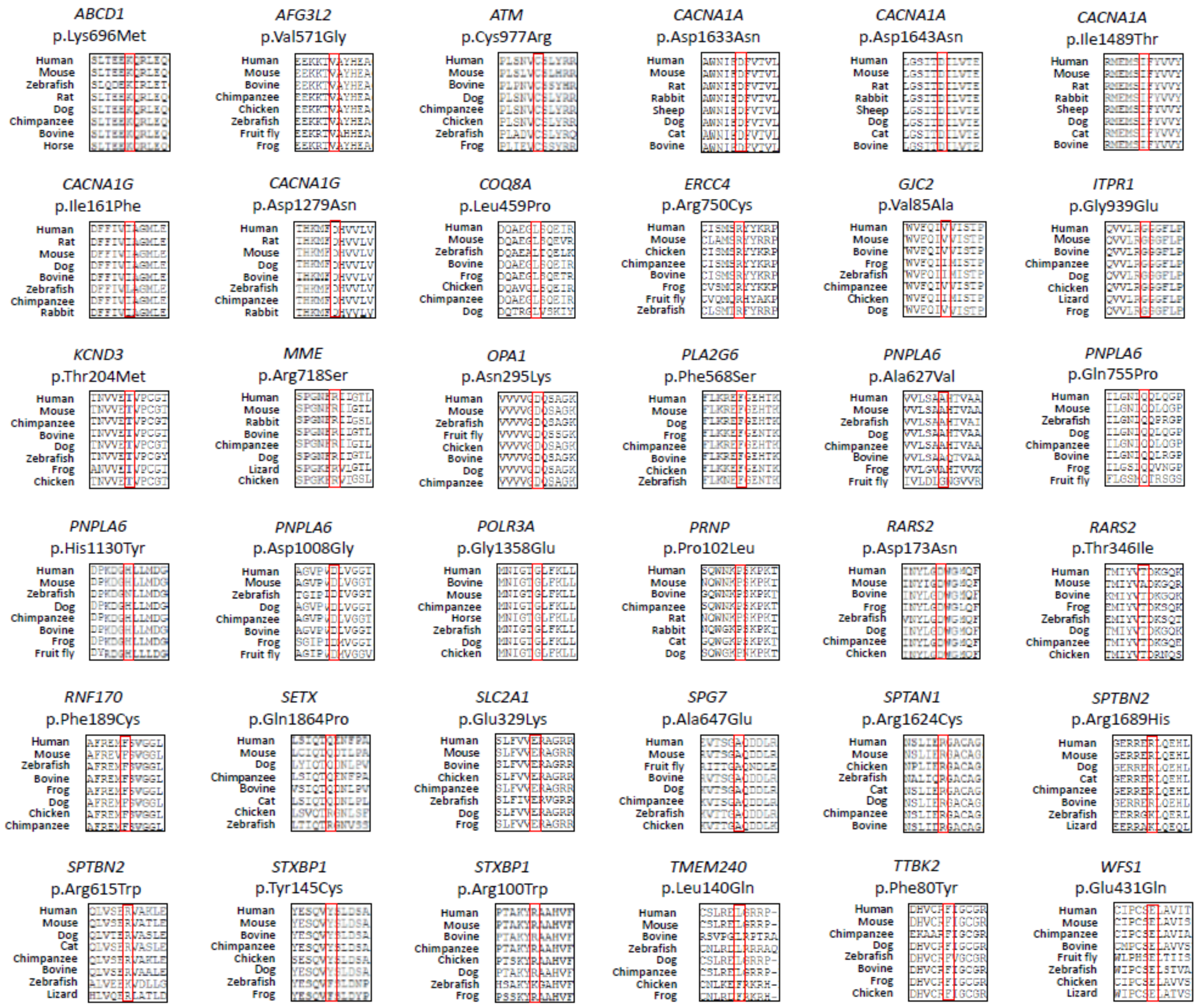

Figure S2. Interatomic interaction changes of missense variants computationally investigated. Wild-type and mutant residues are coloured in light-green.

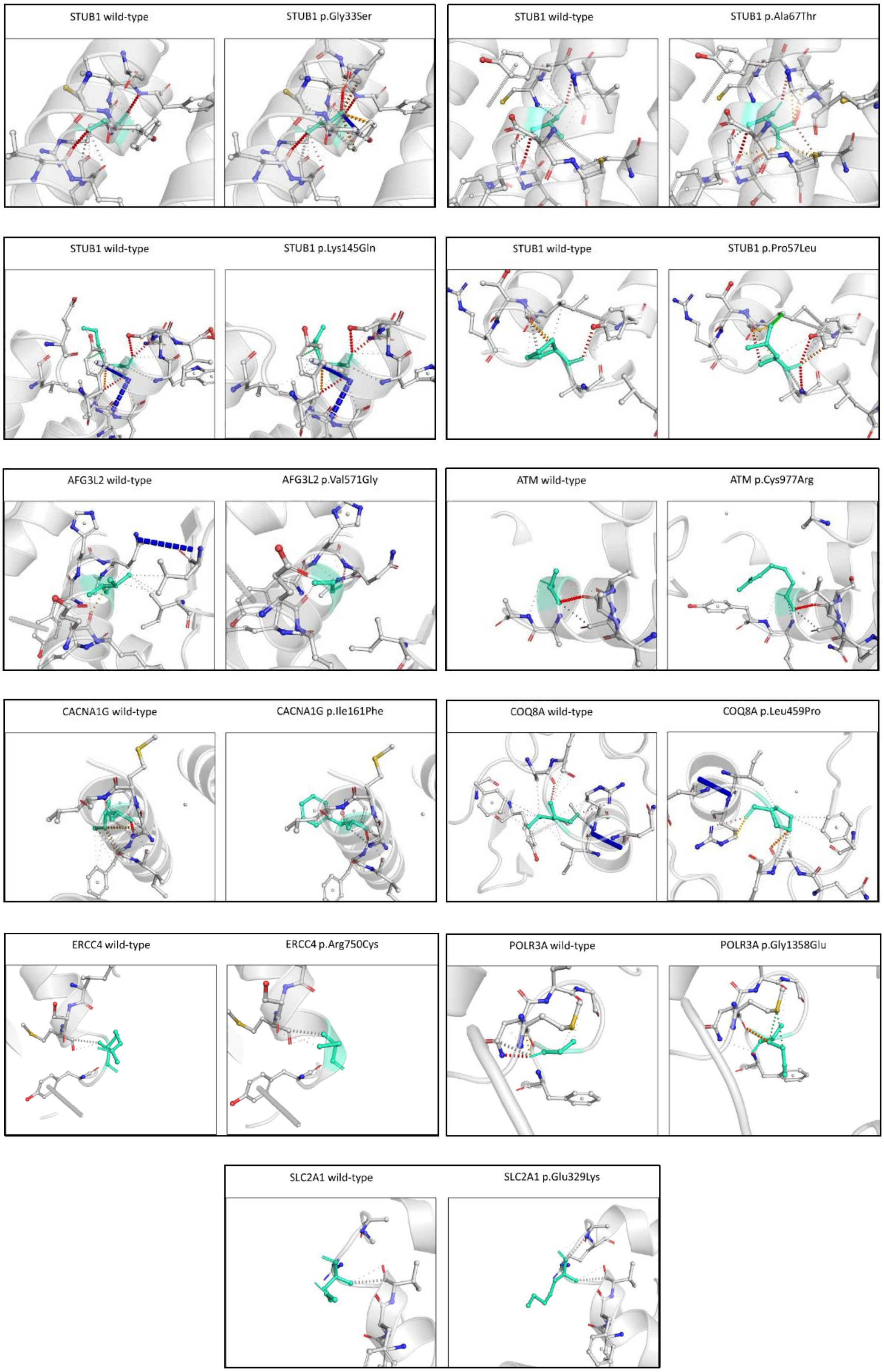

Bond Type	Color	Bond Type	Color
Hydrogen bonds		Ionic interactions	
Water mediated hydrogen bonds		Metal complex interactions	
Weak hydrogen bonds		Aromatic contacts	
Water mediated weak hydrogen bonds		Hydrophobic contacts	
Halogen bonds		Carbonyl contacts	
